# DNA metabarcoding effectively quantifies diatom responses to nutrients in streams

**DOI:** 10.1002/eap.2205

**Published:** 2020-08-18

**Authors:** Nathan J. Smucker, Erik M. Pilgrim, Christopher T. Nietch, John A. Darling, Brent R. Johnson

**Affiliations:** ^1^ Office of Research and Development United States Environmental Protection Agency Cincinnati Ohio 45268 USA; ^2^ Office of Research and Development United States Environmental Protection Agency Research Triangle Park North Carolina 27711 USA

**Keywords:** agriculture, algae, bioassessment, biomonitoring, boosted regression trees, gradient forest, nitrogen, periphyton, phosphorus, rbcL, threshold indicator taxa analysis

## Abstract

Nutrient pollution from human activities remains a common problem facing stream ecosystems. Identifying ecological responses to phosphorus and nitrogen can inform decisions affecting the protection and management of streams and their watersheds. Diatoms are particularly useful because they are a highly diverse group of unicellular algae found in nearly all aquatic environments and are sensitive responders to increased nutrient concentrations. Here, we used DNA metabarcoding of stream diatoms as an approach to quantifying effects of total phosphorus (TP) and total nitrogen (TN). Threshold indicator taxa analysis (TITAN) identified operational taxonomic units (OTUs) that increased or decreased along TP and TN gradients along with nutrient concentrations at which assemblages had substantial changes in the occurrences and relative abundances of OTUs. Boosted regression trees showed that relative abundances of gene sequence reads for OTUs identified by TITAN as low P, high P, low N, or high N diatoms had strong relationships with nutrient concentrations, which provided support for potentially using these groups of diatoms as metrics in monitoring programs. Gradient forest analysis provided complementary information by characterizing multi‐taxa assemblage change using multiple predictors and results from random forest models for each OTU. Collectively, these analyses showed that notable changes in diatom assemblage structure and OTUs began around 20 µg TP/L, low P diatoms decreased substantially and community change points occurred from 75 to 150 µg/L, and high P diatoms became increasingly dominant from 150 to 300 µg/L. Diatoms also responded to TN with large decreases in low N diatoms occurring from 280 to 525 µg TN/L and a transition to dominance by high N diatoms from 525–850 µg/L. These diatom responses to TP and TN could be used to inform protection efforts (i.e., anti‐degradation) and management goals (i.e., nutrient reduction) in streams and watersheds. Our results add to the growing support for using diatom metabarcoding in monitoring programs.

## Introduction

Nutrient pollution from human sources is a widespread problem for water quality and stream ecosystems throughout the United States and around the world (Carpenter et al. 1998, Vörösmarty et al. 2010, United States Environmental Protection Agency 2016). The magnitude and effects of nutrient pollution increase as human populations grow and as watersheds are increasingly altered to produce food and to provide housing and a variety of other socioeconomic and cultural needs (Dodds et al. 2013, Stoddard et al. 2016, Mekonnen and Hoekstra 2018). Increases in nitrogen and phosphorus alter primary producer communities and increase their biomass, which contributes to reduced biodiversity, to changes in food webs and biogeochemical rates, and to negative outcomes in downstream ecosystems (Howarth et al. 2002, Weijters et al. 2009, Woodward et al. 2012, Dodds and Smith 2016). These ecosystem changes can further contribute to reduced recreational opportunities, economic losses, and negative effects on human wellbeing (Dodds et al. 2009, Sobota et al. 2015). Quantifying ecological responses to increasing nutrients is important to addressing these negative effects. Characterizing nutrient–response relationships provides ecological expectations along nutrient gradients and can inform decisions and management practices affecting freshwater resources and their watersheds.

Diatoms are highly responsive to concentrations of nitrogen and phosphorus (Hering et al. 2006, Potapova and Charles 2007, Stevenson 2014), and changes in their assemblage structure have important consequences for primary production and food webs in stream ecosystems. As a result, diatoms increasingly are being used in biological assessments and stream monitoring programs (Danielson et al. 2012, Rimet 2012, Smith et al. 2013, Becker et al. 2019). A variety of diatom species responses to nutrients exist, but assemblage threshold responses along phosphorus and nitrogen concentration gradients are increasingly being reported (Stevenson et al. 2008, Chambers et al. 2012, Smucker et al. 2013*a*, Wagenhoff et al. 2017*a*, Taylor et al. 2018). These threshold responses can be described as large changes in an ecological response occurring across a small change in phosphorus or nitrogen concentrations, as a change in response rates, or as the initiation and cessation of responses (Wagenhoff et al. 2017*b*). Recently developed statistical analyses, such as threshold indicator taxa analysis (Baker and King 2010) and gradient forest analysis (Ellis et al. 2012), are providing new approaches to identifying and summarizing assemblage change along environmental gradients. Characterizing multi‐species assemblage change can provide useful information that complements traditional approaches that aggregate species responses into metrics or indices used in biological assessment and monitoring programs. Regardless of diatom assemblages having threshold or gradual changes, characterizing their nutrient–response relationships could be used to support their use as indicators and to inform potential nutrient targets or criteria development.

Ecological applications of diatoms, such as in bioassessment and for informing nutrient targets, depend on identifying and enumerating species. Morphological characters have been used to describe and identify diatom species since the 1800s, but emerging molecular genetic approaches, such as metabarcoding with high‐throughput DNA sequencing, are becoming increasingly effective for quantifying diatom diversity and relative abundances of gene sequence reads from environmental samples (Apothéloz‐Perret‐Gentil et al. 2017, Vasselon et al. 2017, Rimet et al. 2018). Diatom‐specific primers for the plastid rbcL gene, which is critical to carbon fixation during photosynthesis, are particularly useful for discerning species‐level differences (Kermarrec et al. 2013, Kermarrec et al. 2014). Although databases of diatom rbcL gene sequences are quickly growing, many sequences do not have corresponding taxonomic names and the species coverage is relatively sparse (often <50%) compared to known taxonomic lists (Visco et al. 2015, Cordier et al. 2018). However, this does not preclude the use of DNA metabarcoding in ecological studies because unnamed sequences can be classified as operational taxonomic units (OTUs) and be used in a manner similar to traditional ecological and bioassessment approaches, but with OTU‐based sequence reads instead of morphological count data (Keck et al. 2017, Cordier et al. 2018, Tapolczai et al. 2019).

The use of diatom metabarcoding for environmental assessments and for informing management decisions is in its nascent stage, and the initial focus has been on comparisons to existing diatom indices and estimates of diversity based on microscope identification and enumeration (e.g., Visco et al. 2015, Zimmermann et al. 2015, Apothéloz‐Perret‐Gentil et al. 2017, Vasselon et al. 2017, Pawlowski et al. 2018, Rivera et al. 2018, 2020, Mora et al. 2019, Mortágua et al. 2019). Given the current limitations and uncertainties in reference databases, we examined diatom metabarcoding data using an OTU‐based approach with our goals being to quantify nutrient effects. If effective, possible nutrient–response relationships could inform efforts to protect streams and to manage those experiencing enrichment from human sources. As part of this effort, we also used these data to develop new diatom metrics based on relative abundances of gene sequence reads for OTUs identified by threshold indicator taxa analysis (TITAN) as either increasing or decreasing with TP or TN concentrations. Results from this study can further contribute to understanding the ecology of OTUs identified using DNA metabarcoding.

## Methods

### Study design, sampling, and water chemistry

The East Fork of the Little Miami River watershed located in southwest Ohio, USA is 1,293 km^2^, has a temperate seasonal climate, and has extensive agricultural land use (54%) dominated by soybean and corn production. Streams in this watershed have been routinely monitored for nutrient concentrations since 2008. Based on historical monitoring data, we selected 25 sites in second‐ to third‐order wadeable streams representing gradients of total phosphorus (TP) and total nitrogen (TN). Sites also spanned broad ranges of percent forested, agricultural, and urban land cover in watersheds (Table 1, Appendix S1: Table S1). Streams were sampled for nutrients (*n* = 280) and benthic diatoms (*n* = 342) weekly, 12 times from July through September and once in October 2016. The repeated sampling of sites and subsequent nonparametric statistical analyses with resampling procedures were intended to provide robust characterizations of diatom relationships with nutrients by better capturing conditions within and among sites. The number of samples from each site (13–14 for diatoms and 10–12 for nutrients) ensured similar treatment of all sites in statistical analyses with no one site or group of sites overly influencing results. Ultimately, evenly distributed and continuous gradients of TP and TN concentrations were represented among the streams selected for study (Appendix S1: Fig. S1).

**Table 1:** Descriptive statistics for 25 stream sites.

Parameter	*n*	Minimum	Median	Maximum	Mean ± SD
Watershed area (km^2^)	25	15.8	37.6	82.3	40.3 ± 16.4
Forest (%, 0–100)	25	9	32	59	33 ± 14
Agriculture (%, 0–100)	25	0	53	88	48 ± 29
Urban (%, 0–100)	25	0	8	69	19 ± 20
TP (µg/L)	280	18	171	889	220 ± 179
TN (µg/L)	281	76	620	6,560	776 ± 615
Conductivity (µS/cm)	25	373	544	789	561 ± 112

At each stream site, water for nutrient analyses was collected in a 1‐L acid‐washed polypropylene bottle. Conductivity was not measured during this study, so we used means of historical data for each site to include in data analyses as a proxy of human‐related stressors associated with watershed alteration and because conductivity affects diatoms (Sonneman et al. 2001, Potapova and Charles 2003). Benthic diatoms were collected from five rocks using a firm‐bristled brush attached to a cordless drill within a 6.7 cm^2^ circular guide and composited into one sample. Each rock was collected from five equidistant transects within a 75‐m stream reach. All water samples for nutrient analyses were stored in the dark on ice until returning to the lab where they were stored in the dark at 4°C before being analyzed within 24 h or were stored frozen at −20°C before analysis within 14 d. Nutrient species analyzed included total ammonium, urea, nitrate‐nitrite, total nitrogen, total reactive phosphorus, and total phosphorus. Diatom samples for molecular genetic analyses were frozen until being thawed immediately prior to DNA extraction.

### DNA metabarcoding workflow and bioinformatic analyses

Thawed diatom samples were filtered through sterile, 0.8‐µm polycarbonate filters. The filtered periphyton was subsampled (~50 mg) and ground with a disposable pestle within a 1.5‐mL microcentrifuge tube after exposure to liquid nitrogen. Samples were extracted with Qiagen DNeasy PowerLyzer PowerSoil kits (Germantown, Maryland, USA) following the manufacturer’s instruction, except for an added initial digestion with proteinase K at 56°C for at least 2 h. DNA extractions were quantified using PicoGreen on a BioTek Microplate Reader (Winooski, Vermont, USA) and normalized to 10 ng/µL for polymerase chain reaction (PCR).

DNA extractions underwent PCR to amplify a portion of the chloroplast gene rbcL using previously described primers and reaction conditions (Vasselon et al. 2017). Despite using primers designed to target diatoms, rare nontarget amplification of taxa closely related phylogenetically to diatoms likely cannot be eliminated. PCRs were run as 20‐µL reactions with 2 µL of 10× PCR buffer (with MgCl2), 0.6 µL of 25 mmol/L MgCl2, 1 µL each of the forward and reverse primer cocktails (10 mmol/L; Table 2), 4 µL of 1× BSA, 0.4 µL of 10 mmol/L dNTPs, 0.1 µL of Taq polymerase (Qiagen), 8.9 µL of sterile water, and 2 µL of template DNA. Reaction conditions were 94°C for 150 s, followed by 35 cycles of 94°C for 30 s, 55°C for 1 minute, and 72°C for 1 minute with a final extension of 72°C for 10 minutes. The PCR primers in this round of PCR had 5′ adapter sequences (5′‐ACACTGACGACATGGTTCTACA‐3′ and 5′TACGGTAGCAGAGACTTGGTCT‐3′) for use in the second round of PCR (dual indexing). These PCRs were run in triplicate for each template that was then pooled and cleaned (Qiagen Qiaquick 96 PCR Purification kit) prior to a second PCR where indexing primers were added for sequencing on an Illumina MiSeq (San Diego, California, USA). The second round of PCR had reaction conditions with eight cycles of 95°C for 30 s, 55°C for 30 s, and 72°C for 30 s followed by a final extension step of 72°C for 5 minutes. Index PCR amplicons were then purified using the AMPure XP kit (Beckman Coulter Life Sciences, Indianapolis, Indiana, USA), quantified with PicoGreen as above and normalized in Qiagen EB buffer. Index PCR plates were then pooled into a single sample by combining 3 µL from each well into a 1.5‐mL microcentrifuge tube. Amplicons were then sequenced using a 500‐cycle Illumina MiSeq sequencing kit (2 × 250) according to manufacturer's protocols.

**Table 2:** PCR primer sequences (from Vasselon et al. 2017).

Primer name	Sequence	Cocktail
Diat_rbcL_708F_1	AGGTGAAGTAAAAGGTTCWTACTTAAA	forward
Diat_rbcL_708F_2	AGGTGAAGTTAAAGGTTCWTAYTTAAA	forward
Diat_rbcL_708F_3	AGGTGAAACTAAAGGTTCWTACTTAAA	forward
R3_1	CCTTCTAATTTACCWACWACTG	reverse
R3_2	CCTTCTAATTTACCWACAACAG	reverse

Bioinformatic analyses were performed using USEARCH v9.2 64‐bit (Edgar 2010) on demultiplexed reads from a single MiSeq DNA Sequencing run. Paired reads were merged and primers removed with Cutadapt v 1.14 (Martin 2011). Full‐length sequences that were either shorter than 230 base pairs or had higher than expected errors based on Phred quality scores were excluded. The remainder of the sequences were dereplicated, and unique sequences were identified. Sequences having fewer than four observations in the total sequencing run were excluded to reduce the number of OTUs created by possible sequencing artifacts. The remaining data were screened for chimeric sequences and clustered into Operational Taxonomic Units (OTUs) at ≥97% similarity, a value commonly used and shown to have good discrimination power and performance similar to other thresholds ≥92% (Tapolczai et al. 2019), followed by mapping all the quality‐filtered sequence reads onto these OTUs. The sequence data have been deposited in GenBank (see *Data Availability*).

### Statistical analyses

Relative abundances were used for all statistical analyses (number of rbcL sequence reads for each OTU divided by the sum of sequence reads for all OTUs in a sample). Of 650 OTUs determined in this study, 141 OTUs with ≥1% relative abundance in ≥1% of samples (≥3 out of 342 total) were used in nonmetric multidimensional scaling (NMDS) to examine changes in diatom assemblage structure. For NMDS, we used 50 runs of real data and 1,000 randomizations (PC‐ORD v. 5, MjM Software, Gleneden Beach, Oregon, USA). Prior to the final NMDS ordination, the autopilot mode initially selected three axes for the best solution. Axis 1 was denoted by explaining the most variation. Spearman rank correlations identified relationships among axis scores, diatom metrics, nutrients, and watershed land cover.

**Fig. 1:**
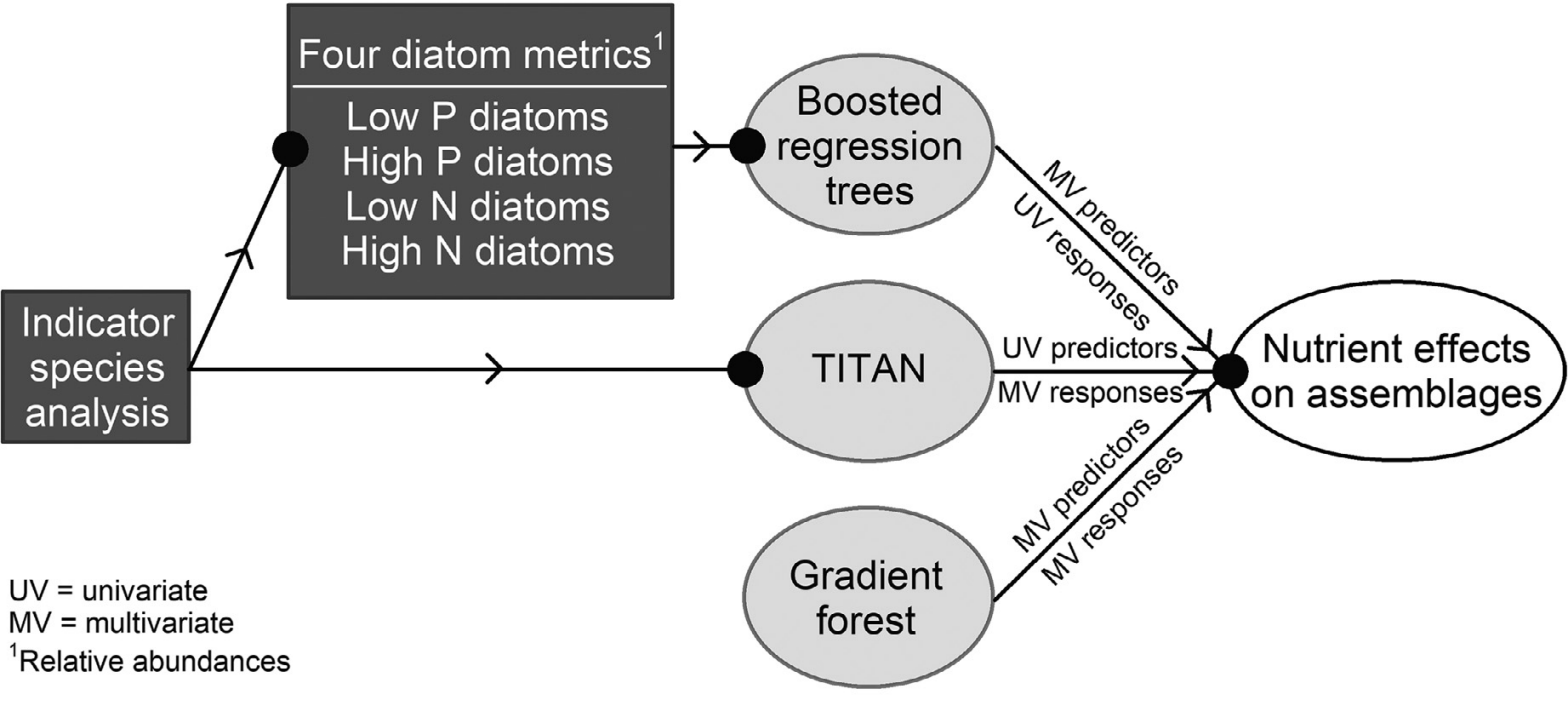
Flow chart showing initial data analyses (dark gray boxes) used in subsequent statistics. Light gray ovals show statistics used to characterize nutrient–response relationships, which were collectively used to summarize diatom assemblage change associated with phosphorus and nitrogen (white oval). Threshold indicator taxa analysis (TITAN) conducts indicator species analysis and designates operational taxonomic units (OTUs) as decreasers, increasers, or unreliable (i.e., not significant) or impure (i.e., inconsistent directionality in responses) based on 1,000 bootstraps. For clarity, we renamed decreasers as low P and low N diatoms and increasers as high P and high N diatoms. Environmental variables were predictors and each diatom metric was subsequently used as a response variable in boosted regression trees (BRTs).

We used R v. 3.4.3 (R Core Team 2017) and the TITAN2 (Baker et al. 2015), dismo (Hijmans et al. 2017), and gradientForest (Ellis et al. 2012) packages to conduct threshold indicator taxa analysis (TITAN), boosted regression tree analyses, and gradient forest analysis, respectively (Appendix S2). These three nonparametric statistical analyses characterized diatom OTU and assemblage relationships with TP and TN concentrations (Fig. 1). We used three analyses because (1) each provides a unique approach to identifying diatom–nutrient relationships, (2) each has been used for informing nutrient targets in other studies (e.g., Smucker et al. 2013*a*, Wagenhoff et al. 2017, Taylor et al. 2018), and (3) each has its strengths and limitations (Fig. 1). TITAN incorporates relative abundance and occurrence data to quantify multi‐taxa assemblage change, but only handles one predictor variable at a time, therefore possibly being confounded by other variables and interactions. Boosted regression trees is a powerful modeling approach that handles multiple predictors, but only one response variable. However, individual response variables, such as diatom metrics, can complement TITAN results, be easy to communicate in a manner similar to traditional biomonitoring, and readily be used in future sampling efforts in the watershed. Gradient forest analysis quantifies multi‐taxa assemblage change and uses multiple predictor variables. Our purpose simply was to use these three statistical analyses as complementary approaches given their growing use and their novel applications to identifying nutrient effects on diatoms using DNA metabarcoding.

TITAN conducts indicator species analysis with bootstrapping to identify the point along an environmental gradient at which each OTU has the greatest change in its frequency and relative abundance (Baker and King 2010). Bootstrapping generates a distribution of change points for each OTU and identifies OTUs with consistently large magnitudes of response (“reliable” with *P* < 0.05) and consistent responses in the same direction as either decreasers or increasers (“purity”) in ≥95% of bootstrap replicates. To facilitate cross‐OTU comparisons, magnitudes of responses are standardized to *z* scores. Assemblage responses are determined by summing *z* scores of all decreaser or increaser OTUs at each partition along an environmental gradient, and the maximum sum *z* score identifies the point at which assemblage change is greatest. Sum *z* scores (sumZ) at each partition of the environmental gradient are presented graphically along with cumulative distributions of bootstrapped change points based on sum *z* scores for decreaser and increaser OTUs. Bootstrapped distributions of OTU change points, shapes of sum *z* score plots, shapes of bootstrapped cumulative frequency distributions of assemblage change points, and how synchronous change points are among OTUs provide information on whether assemblage change has threshold or gradual responses along an environmental gradient (King and Baker 2014). We conducted TITAN for TP and TN gradients with 1,000 bootstraps and included OTUs with at least five observations on either side of a partition (137 OTUs). For clearer communication, we use the terms low or high nutrient diatoms for decreaser and increaser OTUs, respectively. Relative abundances of low P, high P, low N, and high N OTUs were calculated to explore their use as diatom metrics in the manner of traditional bioassessment (e.g., Karr 1981, Kerans and Karr 1994, Hill et al. 2000). If effective, this approach to metric development based on indicator species and diatom metabarcoding could be useful in routine monitoring programs.

To evaluate the use of metrics and to complement the TITAN results based on individual predictors, we used boosted regression trees to examine relationships of diatom metrics and NMDS axis 1 scores with multiple environmental predictor variables. Boosted regression trees use the machine learning method of boosting to build thousands of simple regression trees that are combined to produce a more robust model with higher predictive performance than single tree models (Friedman 2001, Hastie et al. 2009). Boosted regression trees can handle correlated predictors, interactions, and outliers, do not require data transformations, and can model a variety of responses including smooth, curvilinear, or step functions. The relative importance of each variable is determined by randomly adding predictors and identifying how often it is selected and the degree to which it improves the model. Partial dependence plots show fitted functions for each predictor while accounting for mean effects of other variables in the model. The shapes and magnitudes of these plots describe nutrient–response relationships by showing locations along the predictor gradient at which responses occur. Model performance was evaluated using a 10‐fold cross‐validation procedure without replacement that uses 90% and 10% of data for training and validation, respectively (i.e., all data were used for training and prediction steps). Cross‐validation determined mean correlations between fitted and raw values and mean predictive performance of models reported as the percentage of null deviance explained. We conducted boosted regression trees using TP, TN, and conductivity as predictors and each of the four diatom metrics and NMDS axis 1 scores as response variables (Fig. 1). We set tree complexity = 2, learning rate = 0.001, and bag fraction = 0.5, which created more than the recommended minimum of 1,000 trees for each model (Elith et al. 2008).

Gradient forest analysis (Ellis et al. 2012) quantifies changes in assemblage composition by combining results from random forest models for each OTU in a data set (Breiman 2001, Liaw and Wiener 2002). Random forest also combines regression trees with machine learning, can model nonlinear responses, and handle interactions among predictors (Cutler et al. 2007). Each predictor variable is randomly permuted to determine its conditional importance based on the subsequent degradation in model performance. The split values and their importance (i.e., variation explained by the partitioning) from 500 regression trees for each OTU are retained and collated using the R package extendedForest. The R package gradientForest then uses these results to compute multi‐species and assemblage change functions along multiple environmental gradients and to produce visualizations of results (only OTUs with *R* ^2^ > 0 in random forest models were used). Split density plots use the density and weighted importance of OTU splits to identify portions of environmental gradients within which the greatest rates of assemblage change occur. Split densities are standardized to the density of data as a ratio because splits are biased toward portions of gradients with greater sampling density. Standardized split densities with ratios >1 identify regions within which assemblage change is greater relative to elsewhere along the gradient. We interpreted peaks as the predictor values at which greatest change occurred, and the increase and decrease in splits surrounding these peaks as denoting unique regions of change along the gradient when ratios were >1. Cumulative changes of individual OTUs were plotted based on their splits importance distributions and show response curves for each OTU, which were scaled by *R* ^2^ and standardized by data density for better comparison among responses. The overall rate of change in assemblage composition along each environmental gradient was plotted based on averaging the cumulative split importance values for all OTUs.

## Results

TP and TN concentrations for sites were variable over time, site means ranged from 41 to 524 µg TP/L and from 372 to 1,383 µg TN/L, and both were correlated with increasing watershed percent agricultural land cover (Table 3, Appendix S1: Table S1, Fig. S2). In general, TP:TN mass ratios indicated that of the 25 sites, 10 had mean ratios <4, indicating possible N limitation, 13 had mean ratios of 4–10, indicating no limitation or possible co‐limitation, and 2 had mean ratios >10, indicating possible P limitation (Appendix S1: Fig. S3). Total reactive phosphorus to total phosphorus (TRP:TP) ratios varied over time with weekly means ranging from 0.59 to 0.84, and total inorganic nitrogen (mostly NO_3_‐NO_2_) to total nitrogen ratios varied over time and were mostly between 0.36 and 0.59 (Appendix S1: Fig. S4). Two precipitation events were associated with brief increases in TP, TN, and TRP:TP ratios, and in general, TP showed no trend over time whereas TN and TN:TP ratios declined from 3 August to 19 October (Appendix S1: Figs S4–S5).

**Table 3:** Spearman correlations for percent agricultural (Ag) land cover in watersheds, total phosphorus (TP) and total nitrogen (TN) concentrations, nonmetric multidimensional scaling (NMDS) axis 1, and diatom metrics.

Parameter	Ag	TP	TN	NMDS axis 1
High P diatoms	0.59	0.62	0.46	−0.62
Low P diatoms	−0.70	−0.69	−0.47	0.78
High N diatoms	0.44	0.51	0.51	−0.49
Low N diatoms	−0.60	−0.61	−0.61	0.77
NMDS axis 1	−0.76	−0.68	−0.51	
TN	0.43	0.65		
TP	0.76			

Notes

All correlations had *P* < 0.001. Correlations with Ag in watersheds used means of diatom metrics, NMDS axis 1 scores, and nutrients for each site.

**Fig. 2:**
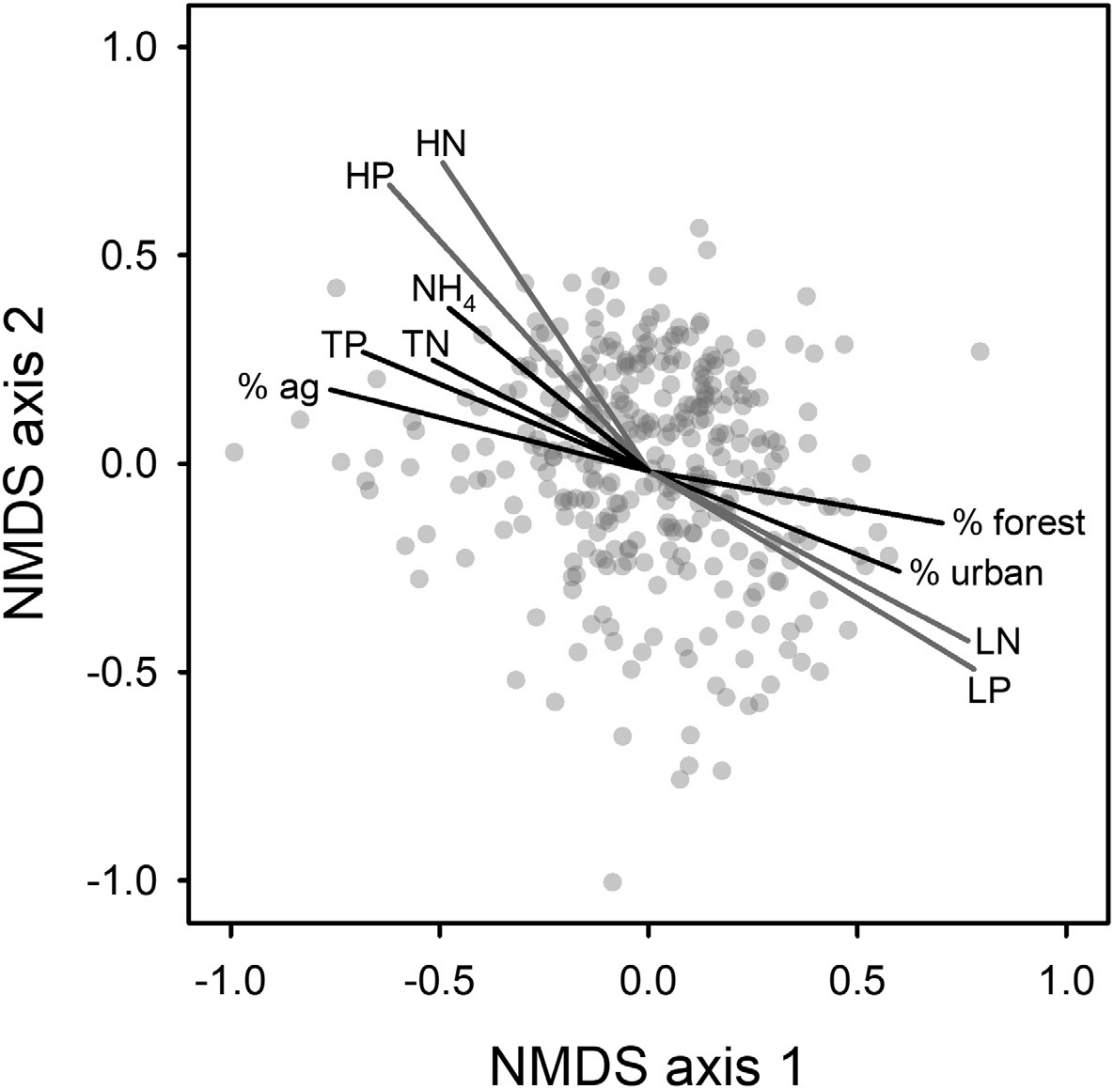
Nonmetric multidimensional scaling (NMDS) ordination of all samples (*n* = 342) using relative abundances of OTU rbcL sequences. Spearman correlation overlays show relationships of axes with land cover, nutrients, and diatom metrics (gray lines). Axes are scaled to coincide with the possible range of correlations from −1 to 1. Ag, agriculture; TP, total phosphorus; TN, total nitrogen; HP, LP, HN, and LN, relative abundances of high and low phosphorus and nitrogen diatoms.

A total of 650 OTUs were observed in the data set of approximately 4.5 million sequence reads (mean per sample = 13,183). OTU richness was 62 ± 22.6 (mean ± SD) and ranged from 14 to 141 per sample, and mean Shannon diversity was 2.6 ± 0.6 and ranged from 0.2 to 4.2 per sample. The three‐dimensional NMDS solution explained 75% of the variance with axis 1 explaining 28.1% and axis 2 explaining 23.5% (stress = 17.5). NMDS axis 1 scores were strongly correlated with agricultural and nutrient gradients (Fig. 2, Table 3, Appendix S1: Table S2). Relative abundances of high P and high N diatom OTUs increased with more negative axis 1 scores, which corresponded with higher nutrient concentrations, whereas those of low P and low N diatom OTUs increased with more positive axis 1 scores and lower nutrient concentrations.

### Diatom responses to TP and TN

**Fig. 3:**
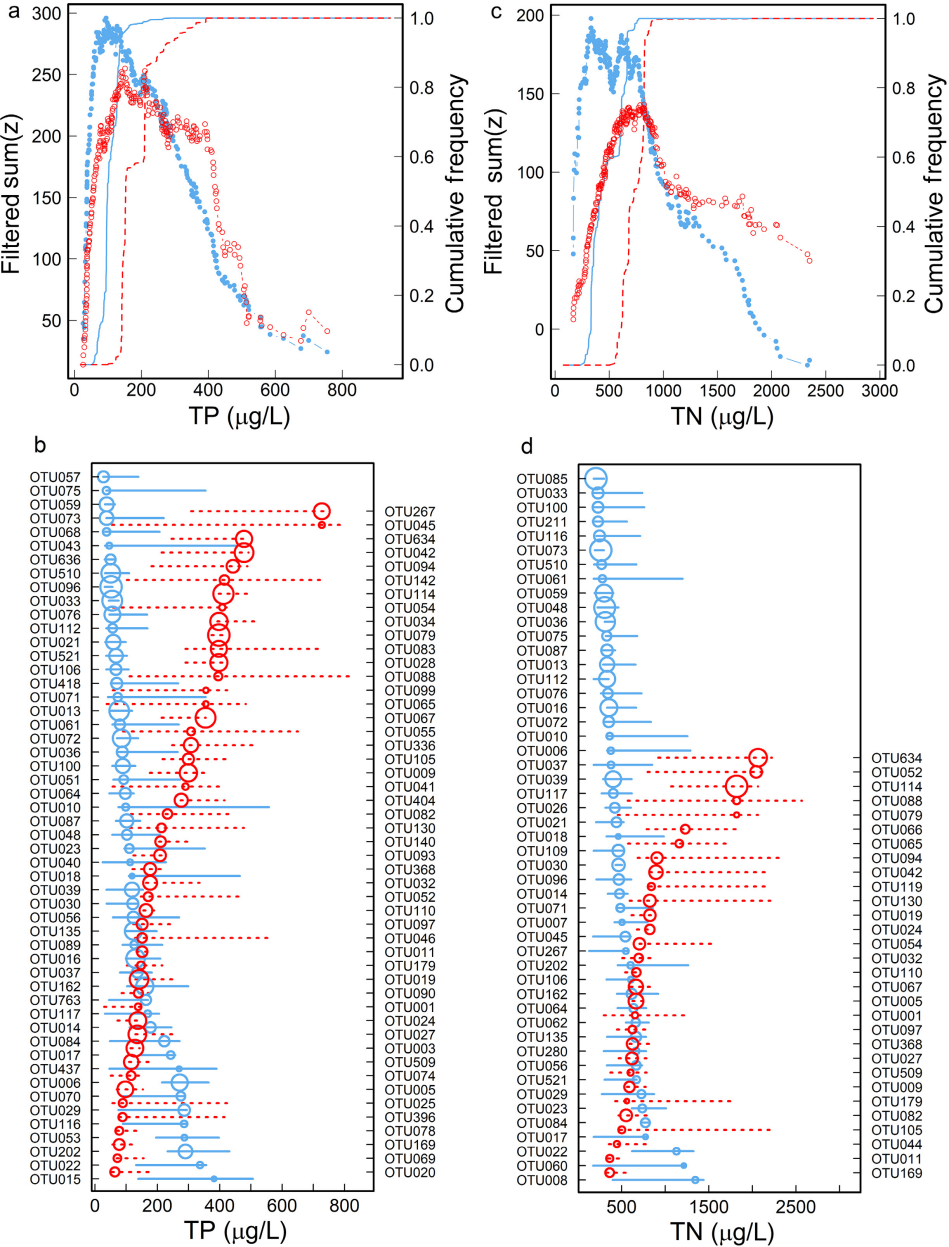
Threshold indicator taxa analysis showing sumZ scores and change points of OTUs for (a, b) TP and (c, d) TN. Filtered refers to results using only OTUs identified as being pure and reliable. Blue shows decreaser sumZ scores and OTUs, red shows increaser sumZ scores and OTUs. In panels a and c. circles show the sumZ scores of decreaser and increaser OTUs at each observed nutrient concentration, and cumulative frequencies show the distribution of assemblage change points (maximum sumZ) from 1,000 bootstraps. In panels b and d, symbols show change points for each OTU scaled according to *z* scores (i.e., magnitudes of responses), and lines show 5th and 95th quantiles from 1,000 bootstraps. Narrow peaks in sumZ scores, steep increases in the cumulative frequency curves, and multiple OTU change points occurring within a narrow range of concentrations provide evidence of thresholds. Broad peaks in sumZ scores (e.g., “plateaus”), gradual increases in cumulative frequency curves, and gradual addition of OTU change points indicate more gradual responses and a longer gradient of assemblage change. The larger maximum sum *z* scores for TP showed that TP had greater effects on diatom assemblage change than did TN.

Of 137 OTUs included in analyses (>1% relative abundance in >1% of samples), TITAN identified 52 as decreasers (low P diatoms) and 49 as increasers (high P diatoms) along the TP gradient (Appendix S1: Table S3). Sum *z* scores for low P diatoms identified an assemblage change point at 96 µg TP/L (5^th^ and 95th percentiles of 68 and 141 µg TP/L), and those for high P diatoms identified a change point at 152 µg TP/L (5^th^ and 95th percentiles of 139 and 310 µg TP/L; Fig. 3a). Cumulative frequency distributions of bootstrapped change points showed that most occurred from 60 to 135 µg TP/L for low P diatoms and from 135 to 160 and 210 to 220 µg TP/L for high P diatoms. For low P diatoms, half of the 52 OTUs had change points between 27 and 102 µg TP/L (Fig. 3b, Appendix S1: Table S3), providing further evidence of substantial assemblage change within this range of TP. Beyond 136 µg TP/L, assemblage change was more gradual as indicated by the linear addition of 15 low P diatom OTU change points. Changes in high P diatoms were more gradual as indicated by a broad peak in sum *z* scores and distributions of 5th–95th percentiles of bootstrapped change points (Fig. 3a), along with the more gradual addition of OTU change points along the TP gradient (Fig. 3b). However, 11 of the 49 OTUs had change points between 128 and 164 µg TP/L, a range that also included the greatest distribution of bootstrapped change points (Fig. 3a).

TITAN identified 50 OTUs as decreasers (low N diatoms) and 30 as increasers (high N diatoms) along the TN gradient (Appendix S1: Table S4). The sum *z* scores for low N diatoms identified an assemblage change point at 333 µg TN/L (5^th^ and 95th percentiles of 307 and 667 µg TN/L), and those for high N diatoms identified a change point at 823 µg TN/L (5^th^ and 95th percentiles of 574 and 851 µg TN/L; Fig. 3c). Cumulative frequency distributions of bootstrapped change points showed that most occurred from 250 to 450 and 560 to 650 µg TN/L for low N diatoms, which also coincided with two peaks in sum *z* scores, and from 560 to 870 µg TN/L for high N diatoms (Fig. 3c). Assemblage response to TN was more gradual than to TP as indicated by a mostly linear addition of low N diatom OTU change points from 206 to 775 µg TN/L, though the magnitude of OTU change (*z* scores) tended to be greatest between 250 and 450 µg TN/L (Figs 3c and d). A more steady increase in sum *z*‐scores and broader distributions of assemblage and OTU bootstrapped change points for high N diatoms further indicated gradual change along the TN gradient.

**Fig. 4:**
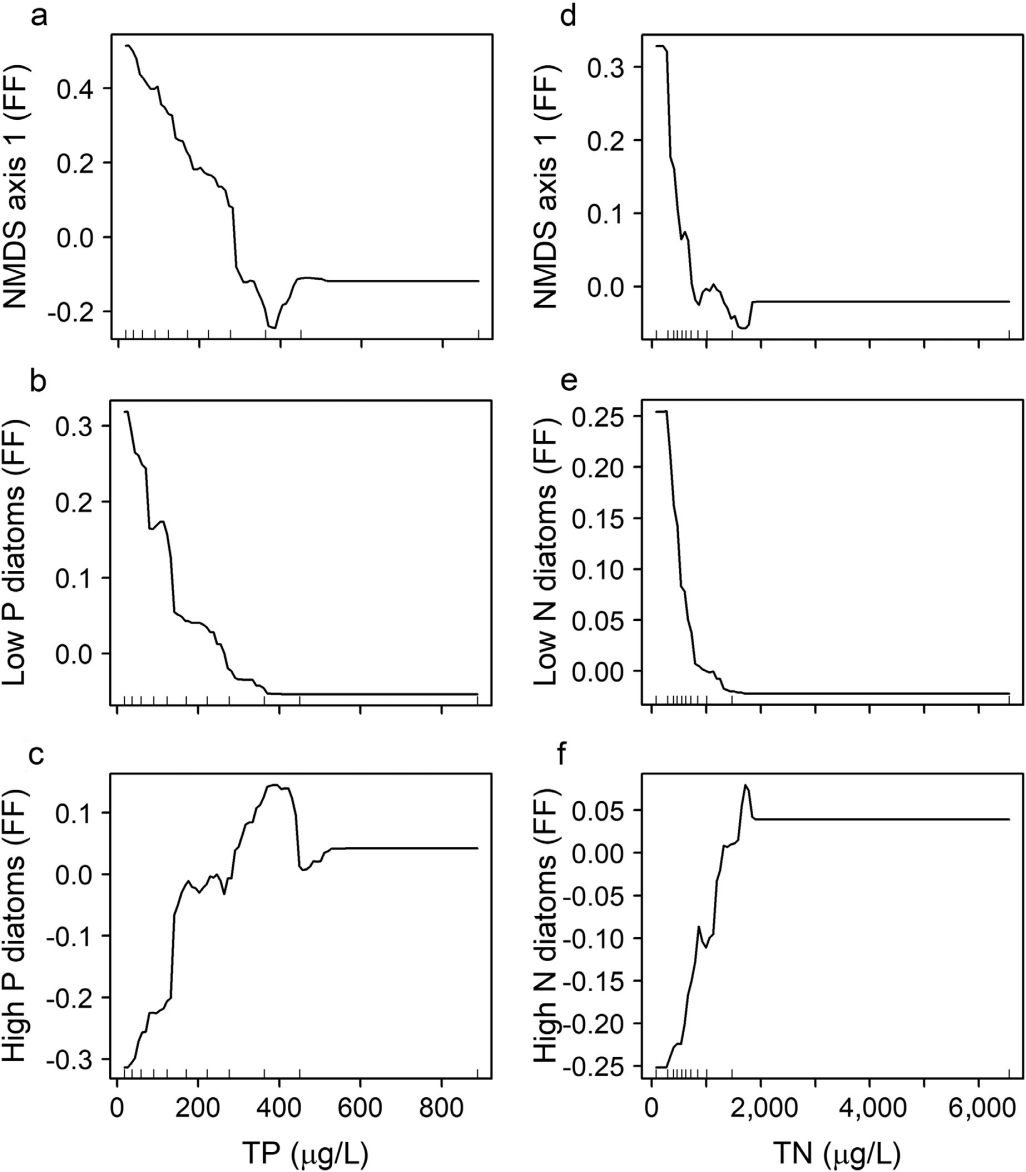
Partial dependence plots from boosted regression trees showing responses of nonmetric multidimensional scaling (NMDS) axis 1 scores and of diatom metrics to TP or TN while controlling for the average effect of other variables. FF, fitted functions. Rug plots show deciles of predictor values.

Boosted regression tree models explained 50–64% of the deviance in diatom metrics and NMDS axis 1 scores, which is how well the models explained observed data (Table 4, Appendix S1: Fig. S6). Cross‐validation showed how well models predicted withheld data, and except for high N diatoms (32%), models performed well and explained 45–49% of the cross‐validated deviance. TP was clearly the most important predictor in NMDS axis 1, low P diatom, and high P diatom models, whereas the importance of TP and TN in low N diatom and high N diatom models were about equal, indicating that TN provided substantial and unique contributions to these diatom responses. Partial dependence plots showed multiple points along TP and TN gradients at which diatom metrics had large responses and showed concentrations beyond which responses no longer decreased or increased (Fig. 4). NMDS axis 1 scores showed gradual assemblage change as they declined linearly from 28 to 185 µg TP/L, but they also had a steep decline from 283 to 308 µg TP/L. Low P diatoms had steep and increasingly large declines beginning at 32, 74, and 118 µg TP/L before having a gradual decline from 207 to 293 µg TP/L. High P diatoms had a small gradual increase from 25 to 80 µg TP/L, a large increase from 129 to 175 µg TP/L, and a final small increase from 283 to 290 µg TP/L prior to a large hump in the response curve. Low N diatoms had a large decrease from 281 to 531 µg TN/L and a modest decrease from 594 to 788 µg TN/L. High N diatoms had large increases from 538 to 850 and from 1,212 to 1,344 µg TN/L.

**Table 4:** Results from boosted regression tree models for nonmetric multidimensional scaling axis 1 scores and for each diatom metric showing deviance explained as a percentage of the null deviance for observed data and of deviance explained using cross‐validated (CV) data (i.e., 10‐fold cross‐validation of data withheld to examine predictive performance), CV correlations between raw and fitted values, and relative importance of predictor variables.

Response	Observed deviance explained (%)	CV deviance explained (%)	CV correlation	Relative importance (%)		
				TP	TN	Conductivity
NMDS axis 1	61	48 ± 9	0.72 ± 0.04	52	15	32
Low P diatoms	55	45 ± 4	0.69 ± 0.03	74	9	17
High P diatoms	64	49 ± 5	0.71 ± 0.03	57	19	24
Low N diatom	61	48 ± 5	0.70 ± 0.03	44	36	20
High N diatoms	50	32 ± 7	0.60 ± 0.03	39	40	21

Note

Mean ± SE is presented for CV values.

**Fig. 5:**
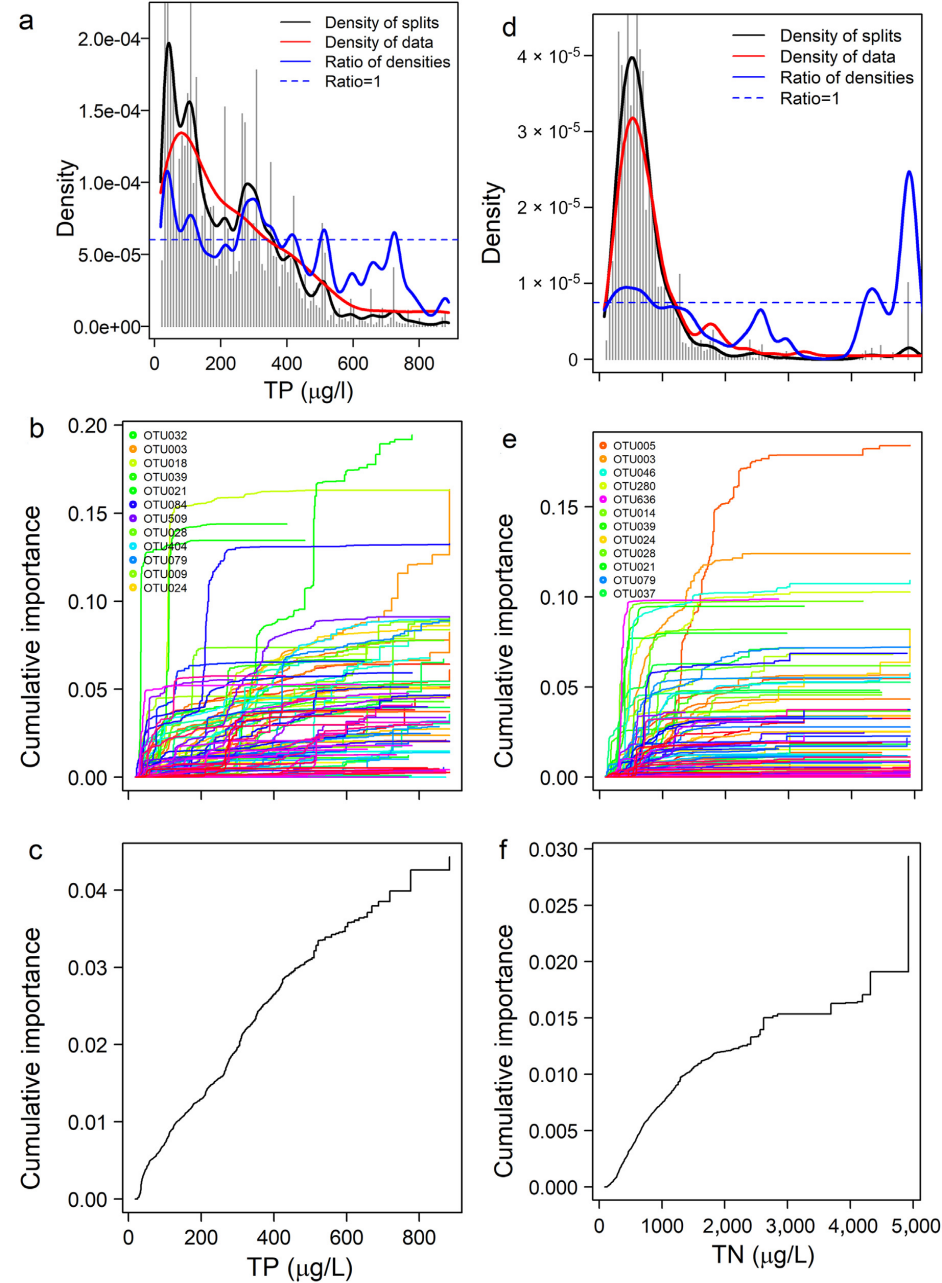
Results from gradient forest analysis showing diatom OTUs and assemblage responses to TP and TN. (a) Compositional change based on aggregating OTU responses were determined by split importance and values along TP and TN gradients (bars) and the ratios (blue lines) of split density (black lines) to data density (red lines). Peaks and regions of standardized split density plots with ratios >1 (horizontal dashed lines) delineate portions of the TN or TP gradient within which OTU compositional change is relatively greater than elsewhere along the nutrient gradient. (b) Cumulative change of individual OTUs is shown based on their splits’ importance distributions, which were scaled by *R* ^2^ and standardized by data density for each OTU with the most important OTUs being labeled. (c) Relative rates of overall change in assemblage composition based on cumulative splits importance plots along the TP and TN gradients.

Gradient forest analysis identified 99 diatom OTUs with *R* ^2^ values ranging from 0.002 to 0.614 (mean = 0.14) with mean *R* ^2^ values of the top 50 OTUs and bottom 49 being 0.23 and 0.06, respectively (Fig. 5, Appendix S1: Table S5, Fig. S7). Peaks in the standardized split density plot showed that the greatest changes in diatom assemblages occurred from 20–75, from 75 to 134, and from 250 to 367 µg TP/L, with smaller peaks indicating additional, albeit more minor, changes at 418, 513, and 722 µg TP/L (Fig. 5a). These peaks corresponded with portions of the TP gradient within which steep increases in cumulative importance occurred for several diatom OTUs (Fig. 5b) and with relatively steeper increases in the plots showing the cumulative change in assemblage composition (Figs. 5c), which represents average OTU change. Assemblage change occurred more gradually along the TN gradient between 153–833 µg TN/L (Fig. 5d), which encompassed the majority of change in the cumulative importance for most OTUs and assemblage change (Fig. 5e–f). The peaks at 4,362 and 4,875 µg TN/L likely were artefacts of low data density combined with relatively minor changes in OTU024.

### Summary of diatom responses

**Fig. 6:**
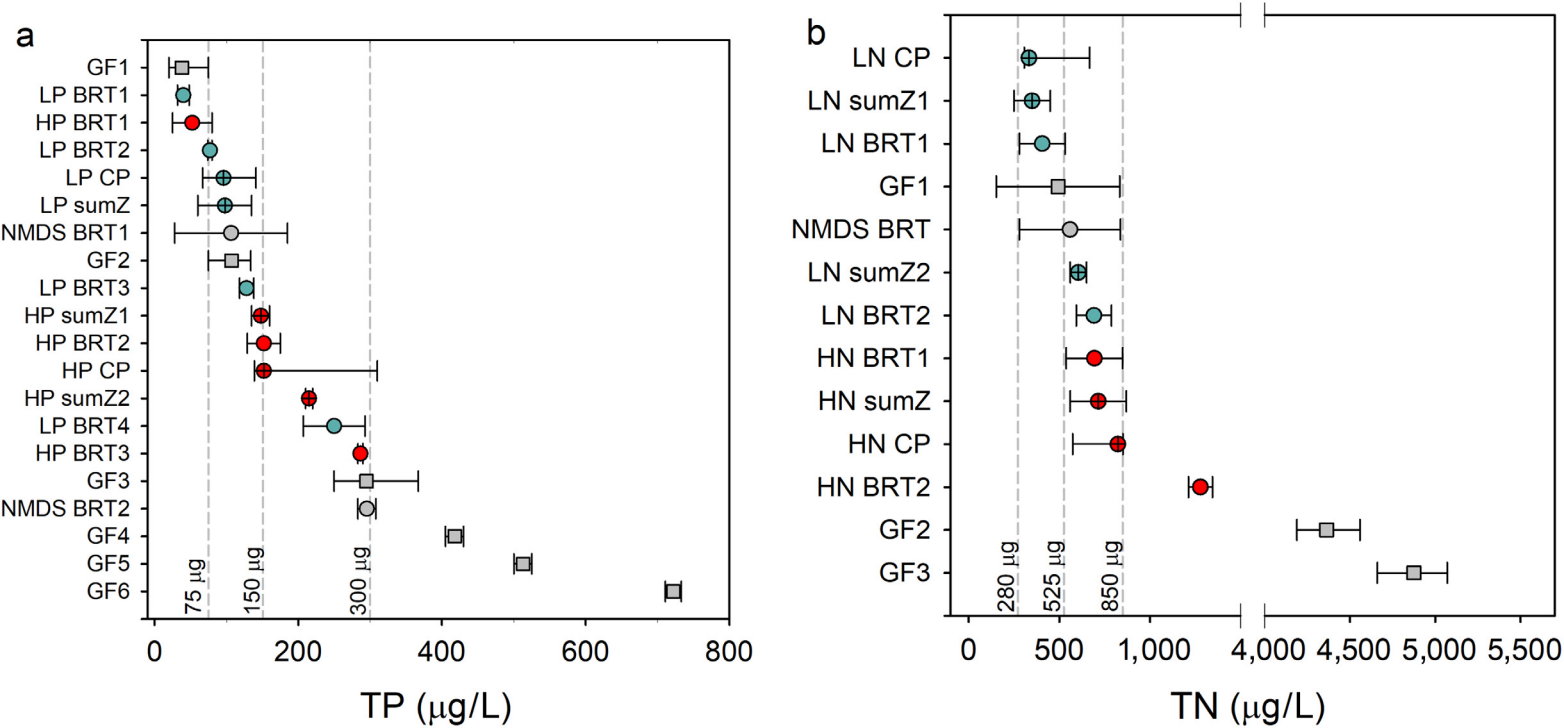
Summaries of diatom OTU responses to (a) total phosphorus and (b) total nitrogen. In panel b, *x*‐axis break is 1,499–3,999 µg/L. Crossed circles are results directly from TITAN (change points [CP] with bootstrapped 5th and 95th quantiles and mid‐points in the portions of cumulative frequency distributions showing the greatest increases in bootstrapped change points). Circles are boosted regression tree (BRT) results showing mid‐points from portions of partial dependence plots with substantial change in response variables. Squares are peaks in standardized splits density plots in gradient forest (GF) analysis. Colors denote responses by low nutrient diatom OTUs (blue), high nutrient diatom OTUs (red), and assemblage change (gray; GF and nonmetric multidimensional scaling [NMDS]). Horizontal lines represent the range within which notable responses occurred (or 5th and 95th quantiles of TITAN change points). Diatom responses occurred at multiple points along the TP or TN gradient and these are denoted as BRT1, BRT2…, GF1, GF2…, and sumZ1, sumZ2. Vertical dashed gray lines demarcate portions of TP and TN gradients within which substantial changes in diatom assemblage occurred (see Summary of diatom responses in Results).

The multiple statistical analyses identified strong relationships between diatoms and increasing concentrations of TP and TN. Less complex univariate analyses showed similar trends as well (Appendix S1: Fig. S8). While there was within‐site variability in diatom metrics and NMDS axis 1 scores during the study (Appendix S1: Fig. S9), their relationships with nutrient concentrations were strong (Figs. 2, 3, 4, 5, Appendix S1: Fig. S10). A synthesis of results from TITAN, boosted regression trees, and gradient forest analysis showed that multiple nutrient concentrations denoted important portions of the TP and TN gradients where large changes in diatom assemblages occurred (Fig. 6, Appendix S1: Tables S6–S7). In general, diatom OTUs and assemblages began changing from approximately 20–75 µg TP/L, as indicated by the largest peak in the split density plot, low P diatom change points, and a small yet notable decrease in the relative abundance of low P diatoms. The TITAN change point for low P diatoms (96 µg TP/L), large decreases in relative abundances of low P diatoms, and another peak in split densities occurred from 75 to 150 µg TP/L. From 150 to 300 µg TP/L, the TITAN change point for high P diatoms occurred (152 µg TP/L), high P diatoms had large increases in relative abundances, and the final decrease in low P diatom relative abundances was observed. Above 300 µg TP/L, diatom assemblages were substantially altered and only a few additional minor changes occurred based on gradient forest analysis and OTU change points in TITAN.

In general, diatom assemblages had gradual but substantial changes from approximately 280 to 850 µg TN/L (Fig. 6). The TITAN change point for low N diatoms (333 µg TN/L) and the largest decrease in relative abundances of low N diatoms occurred from 280 to 525 µg TN/L. Mid‐points of overall assemblage change occurred near 525 µg TN/L (e.g., peak in split density plot and middle of NMDS1 response in boosted regression trees). From 525 to 850 µg TN/L, high N diatoms had large increases in relative abundances, the final decrease in low N diatom relative abundances was observed, and the TITAN change point for high N diatoms occurred (823 µg TN/L). Above 850 µg TN/L, only minor changes in diatom assemblages occurred aside from a second increase in high N diatom relative abundances from 1,212 to 1,344 µg TN/L.

## Discussion

### Diatom DNA metabarcoding

Multiple statistical analyses identified large changes in assemblage structure associated with increasing TP and TN concentrations—providing strong support for using diatom metabarcoding in monitoring programs and for developing responsive indicators. The sensitivity of diatom assemblages was particularly evident from the high proportions of OTUs identified as significant responders to TP and TN in TITAN (101 of 137 and 80 of 137, respectively) and in the gradient forest analysis (99 of 137). Furthermore, the statistical models performed similarly well when compared to those from other studies using morphological identification and enumeration of diatoms or algae (Smucker et al. 2013a, 2013b, *2013a*, *2013b*, Wagenhoff et al. 2017, Munn et al. 2018, Taylor et al. 2018). Our application of metabarcoding contributes to the small, but growing, body of evidence that gene sequence‐based diatom monitoring provides useful ecological information and can be used in a manner similar to existing morphology‐based approaches to environmental assessments and water quality criteria development (Cordier et al. 2018, Pawlowski et al. 2018).

Applications of metabarcoding approaches in biomonitoring contexts historically have faced a variety of challenges, such as PCR bias, sequencing error, uncertainty in sequence‐based abundance estimation and taxonomic assignments, and the need for optimization and standardization of molecular and bioinformatics workflows. However, existing methods and analytical tools reduce their effects and will continue improving as rapid advancements in technology and data analysis occur (Keck et al. 2017). Although using molecular‐based relative abundances is common, comparisons with morphological‐based identification and enumeration can be problematic (though see references for examples of comparisons with diatom indices and diversity). Numbers of gene copies per cell can be variable among species, can increase with greater biovolume, and can be affected by environmental variables and the physiological condition of diatoms (Vasselon et al. 2018, Vasselon et al. 2019). Further complicating comparisons between molecular‐ and morphological‐based relative abundances are other known issues, such as sparse taxonomic coverage in bioinformatic libraries, possible cryptic species being missed in microscope counts, differences in morphological identification and enumeration among taxonomists and laboratory methods (Kelly 1997, Kahlert et al. 2012, Lavoie and Campeau 2016), and dead cells typically being included in diatom counts (Gillett et al. 2011). Regardless of the validity of comparisons to morphological‐based approaches, our study effectively identified relationships between diatom assemblages and concentrations of phosphorus and nitrogen using relative abundances of unique sequences in an OTU‐based approach without taxonomic identification.

The capability to match rbcL sequences with taxonomic names remains limited, and this hinders the ability to connect new molecular genetic data to the rich history of diatom research, biogeography, and ecology (e.g., Patrick 1961, Lowe 1974, Lange‐Bertalot 1979, Smol and Stoermer 2010). Most sequences in our data set did not have 100% bootstrap support for assignment to species names in available taxonomic databases, which indicated limited confidence in assignment accuracy, and many sequences in reference databases had no species name (Appendix S1: Tables S3–S5), further highlighting this taxonomic limitation. The taxonomic assignments in Appendix S1 should therefore be considered preliminary, and we simply provide them in the supplement for interested readers. Reference barcode libraries are quickly growing (e.g., Rimet et al. 2016) and should continue improving links to historical diatom monitoring, indices, and research. In the interim, given the current limitations and uncertainties in reference databases, ongoing applications of diatom metabarcoding in a biomonitoring context simply could continue to adopt taxonomy free approaches (Apothéloz‐Perret‐Gentil et al. 2017, Cordier et al. 2018). The ecological understanding of gene sequence‐based OTUs will further improve as more research characterizes their relationships with environmental conditions.

### Diatom responses to nutrients

In our study watershed, diatom assemblages significantly changed with increasing nutrient concentrations, which were associated with greater amounts of agriculture in upstream watersheds. Although TN:TP mass ratios suggested that N limitation may be more common in this watershed, gradient forest analysis, NMDS axis 1 responses in boosted regression trees, and sum *z* scores in TITAN indicated that diatom assemblages and OTUs had stronger relationships with TP than with TN. However, TN still was an important predictor in TITAN, explained additional unique variation in gradient forest analysis, and was particularly important in boosted regression tree models for low N and high N diatom metrics. Gradient forest analysis and boosted regression tree models also showed different relationships with TP and TN for OTUs, for assemblage change, and for low and high P or N diatom metrics. These differences supported distinct inferences from TP and TN and indicated that diatom responses were not simply inverse relationships between low and high N or P diatoms nor were diatom responses to TP similar to responses to TN, even if certain OTUs increased or decreased with both. Despite phosphorus or nitrogen being capable of individually dominating the effects on stream primary production and algal assemblage structure (e.g., Stevenson et al. 2008, Smucker et al. 2013*a*, Taylor et al. 2018), our results showed that a second nutrient can explain additional changes as well. Being able to discern these separate effects would be particularly useful for monitoring programs and management efforts in watersheds, states, and regions where streams could be P‐, N‐, or co‐limited, which are common situations (Francoeur 2001, Hill et al. 2012) and occurred in our study watershed.

Most changes in diatom assemblages and OTUs occurred between approximately 150–850 µg TN/L and 20–150 µg TP/L, but subsequent noteworthy changes also occurred from 150 to 300 µg TP/L and several OTUs had TITAN change points above 400 µg/L. These TN concentrations span oligotrophic to eutrophic conditions (Dodds 2007) and include ranges of concentrations within which diatom responses have been reported in the literature (Chambers et al. 2012, Lavoie et al. 2014, Hausmann et al. 2016). The responses at higher TP concentrations and in more heavily agricultural watersheds tended to be greater than those commonly reported. Other studies have found diatom threshold responses at concentrations typically ranging from 10 to 80 µg TP/L (Stevenson et al. 2008, Chambers et al. 2012, Smucker et al. 2013*a*, Taylor et al. 2018). Minimal change in assemblages (i.e., response cessation) above these concentrations has been reported, potentially limiting diatom applications in streams with higher nutrient concentrations or intensities of watershed agriculture (Waite 2014, Pillsbury et al. 2019). However, many diatom species are indicators of TP concentrations >100 µg/L (Potapova and Charles 2007), suggesting that additional changes in taxa and assemblage structure in high nutrient systems could be expected. Though less common, some studies have reported changes in diatom species and assemblages at higher TP (up to 156 µg TP/L) and TN (up to 1,100 µg TN/L) concentrations (Smith and Tran 2010, Chambers et al. 2012, Lavoie et al. 2014, Hausmann et al. 2016, Taylor et al. 2018, Hicks and Taylor 2019).

Reported concentrations at which thresholds occur or at which responses begin and end could be affected by confounding factors associated with differences in (1) regional background nutrient concentrations, (2) current and past land use, (3) statistical analyses, (4) the spatial extent and design of surveys, and (5) how diatom responses are summarized (e.g., various metrics versus multi‐taxa assemblage responses). We sought to minimize effects of confounding factors by specifically targeting an evenly distributed and continuous nutrient gradient in similarly sized streams within a large watershed. We similarly examined multiple ways of summarizing and analyzing diatom responses to overcome potential limitations associated with any single method and to compare results among them. The multiple statistical analyses and diatom responses provided complementary details on how diatom assemblages changed as nutrient concentrations increased (Fig. 6). In general, the transitions from low to high P or N diatom dominance likely result from differences in species‐specific growth rates. Experiments have found that growth rates of low nutrient taxa increase more slowly with increasing nutrient concentrations and their maximal growth rates are slower and occur at lower nutrient concentrations than those for high nutrient diatom species (Manoylov and Stevenson 2006, Shatwell et al. 2014). Collectively, the combined results provided support for developing diatom indicators using DNA metabarcoding that are responsive to nutrient effects on stream ecosystems and that could inform management decisions.

## Conclusions

Algal assemblages are increasingly being recognized as sensitive responders to nutrient pollution that would be useful in monitoring programs and criteria development (United States Environmental Protection Agency 2014). Our results demonstrated that DNA metabarcoding of stream diatoms can be a useful new approach to quantifying nutrient effects on streams, possibly even in heavily agricultural areas or in watersheds spanning broad N or P gradients as in our study. Gradient forest analysis and TITAN provided powerful analyses of multi‐taxa assemblage changes and were useful for identifying important regions of change along TP and TN gradients. Not only were assemblages strong responders to increasing TP and TN concentrations, but the low and high P and N diatom metrics based on TITAN results effectively summarized OTU responses, and their responses in partial dependence plots closely coincided with output from gradient forest analysis and TITAN in most instances. This suggests that diatom metrics using relative abundances of gene sequence reads could be readily incorporated into monitoring programs. The applications of diatom metabarcoding to identifying stressor effects will continue benefiting from additional work at a variety of spatial extents and in other regions. Diatom responses like those observed here also could have potential for informing the development of nutrient criteria and decisions regarding discharge permits, land use, and best management practices that target the reduction of nutrient loads to streams. Ongoing improvements in methods, technology, and data analysis, along with examples of useful applications, such as those in our study, can promote the use of diatom metabarcoding as a relatively quick and cost‐effective approach used in environmental assessments and monitoring programs.

## Supporting information

Appendix S1

Appendix S2

## Data Availability

Data are available in the United States Environmental Protection Agency's ScienceHub repository: https://doi.org/10.23719/1504034. Molecular genetic data are available in GenBank under BioProject ID PRJNA592969 (http://www.ncbi.nlm.nih.gov/bioproject/592969).
