# Supplementary material for: DNA metabarcoding effectively quantifies diatom responses to nutrients in streams
Source: Ecol Appl. 2020 Aug 18;30(8):e02205. doi: 10.1002/eap.2205 (PMC7731896; doi:10.1002/eap.2205)
Supplement: Supplementary file 1 — Appendix S1 [file EAP-30-e02205-s001.pdf]

**Supporting Information.** Smucker, N.J., E.M. Pilgrim, C.T. Nietch, J.A. Darling, and B.R. Johnson. 2020. DNA metabarcoding effectively quantifies diatom responses to nutrients in streams. Ecological Applications.

Appendix S1

Table S1. Site information, watershed land cover, mean nutrient concentrations, and mean diatom values for each site. Number of diatom samples and nutrient samples per site were 13–14 and 10–12, respectively.

| Site | Latitude | Longitude | Drainage area ($km^2$) | Agriculture (%) | Urban (%) | Forest (%) | TP ppb | TN ppb | NMDS1 | NMDS2 | HP diatoms (RA) | LP diatoms (RA) | HN diatoms (RA) | LN diatoms (RA) |
|---|---|---|---|---|---|---|---|---|---|---|---|---|---|---|
| 890 | 39.20434 | -83.76418 | 32.87 | 88.0 | 0.0 | 12.0 | 503 | 954 | -0.472 | 0.644 | 0.79 | 0.03 | 0.56 | 0.08 |
| BBC | 39.08956 | -84.16902 | 50.32 | 51.9 | 9.5 | 38.4 | 129 | 484 | 0.233 | -0.384 | 0.18 | 0.47 | 0.12 | 0.56 |
| BRF | 39.13871 | -84.13162 | 76.51 | 53.3 | 5.0 | 41.0 | 159 | 758 | 0.196 | -0.335 | 0.16 | 0.32 | 0.13 | 0.44 |
| FMC | 39.09732 | -84.02619 | 37.55 | 64.3 | 5.2 | 30.4 | 314 | 756 | -0.086 | 0.424 | 0.72 | 0.11 | 0.55 | 0.14 |
| FMR | 39.05470 | -84.16780 | 26.45 | 27.0 | 31.5 | 40.4 | 62 | 414 | 0.302 | -0.566 | 0.12 | 0.50 | 0.11 | 0.50 |
| FVC | 39.11360 | -84.02030 | 70.54 | 76.2 | 5.6 | 18.2 | 392 | 997 | -1.259 | 0.159 | 0.77 | 0.05 | 0.72 | 0.09 |
| FVM | 39.10728 | -83.93254 | 22.01 | 85.0 | 6.0 | 9.0 | 524 | 991 | -0.455 | -0.127 | 0.40 | 0.08 | 0.32 | 0.22 |
| GRR | 39.13293 | -84.01522 | 41.70 | 73.7 | 3.7 | 22.5 | 215 | 813 | -0.005 | 0.491 | 0.55 | 0.17 | 0.44 | 0.29 |
| HLR | 39.14030 | -84.25920 | 34.84 | 5.3 | 62.6 | 32.1 | 139 | 922 | 0.412 | -0.767 | 0.10 | 0.52 | 0.11 | 0.54 |
| HWR | 39.12400 | -84.00733 | 36.22 | 78.5 | 3.8 | 17.6 | 366 | 908 | -0.476 | 0.003 | 0.56 | 0.10 | 0.32 | 0.15 |
| KAR | 39.03330 | -84.08190 | 41.46 | 53.5 | 11.6 | 34.7 | 98 | 537 | 0.132 | -0.395 | 0.18 | 0.49 | 0.14 | 0.51 |
| LCF | 39.14723 | -84.17835 | 42.61 | 31.7 | 12.4 | 55.3 | 176 | 525 | 0.391 | 0.019 | 0.22 | 0.38 | 0.18 | 0.37 |
| LRC | 39.05970 | -84.18060 | 49.66 | 25.1 | 28.6 | 46.1 | 121 | 536 | 0.091 | -0.042 | 0.35 | 0.27 | 0.24 | 0.29 |
| LRN | 38.99300 | -84.05900 | 31.06 | 48.1 | 4.6 | 47.1 | 86 | 615 | 0.357 | -0.127 | 0.18 | 0.46 | 0.17 | 0.52 |
| MOF | 39.19770 | -84.06076 | 26.09 | 74.4 | 4.0 | 21.5 | 363 | 1023 | -0.346 | 0.081 | 0.42 | 0.11 | 0.39 | 0.19 |
| PLR | 39.11250 | -84.04220 | 47.18 | 70.8 | 3.8 | 24.9 | 355 | 958 | -0.244 | 0.048 | 0.54 | 0.11 | 0.37 | 0.19 |
| S1 | 39.13470 | -84.23250 | 24.30 | 9.7 | 34.1 | 56.1 | 73 | 468 | 0.611 | 0.239 | 0.23 | 0.44 | 0.23 | 0.49 |
| SAR | 39.13720 | -84.24580 | 42.27 | 10.9 | 40.4 | 48.2 | 71 | 394 | 0.710 | -0.121 | 0.18 | 0.57 | 0.16 | 0.59 |
| SHO | 39.08830 | -84.22170 | 34.87 | 14.7 | 48.2 | 36.7 | 90 | 563 | 0.279 | 0.069 | 0.36 | 0.40 | 0.32 | 0.32 |
| SHR | 39.11780 | -84.21640 | 82.29 | 11.5 | 48.6 | 39.5 | 58 | 372 | 0.509 | -0.448 | 0.14 | 0.53 | 0.14 | 0.53 |
| SOR | 39.20220 | -83.90970 | 41.73 | 77.6 | 6.1 | 16.1 | 402 | 1028 | -0.751 | -0.046 | 0.66 | 0.10 | 0.43 | 0.10 |
| SYC | 39.20580 | -83.94500 | 43.58 | 71.7 | 3.9 | 24.4 | 214 | 957 | -0.652 | 0.192 | 0.55 | 0.11 | 0.42 | 0.18 |
| ULR | 39.00215 | -84.15147 | 23.75 | 17.9 | 23.3 | 58.8 | 254 | 1383 | 0.145 | 0.516 | 0.59 | 0.20 | 0.56 | 0.20 |
| USR | 39.11710 | -84.25926 | 15.79 | 0.0 | 68.8 | 31.2 | 41 | 463 | 0.483 | 0.166 | 0.17 | 0.43 | 0.17 | 0.46 |
| UST | 39.25640 | -84.01250 | 30.95 | 77.32 | 8.21 | 13.80 | 199 | 947 | -0.170 | 0.283 | 0.59 | 0.16 | 0.52 | 0.15 |

Table S2. Spearman correlations of nonmetric multidimensional scaling axes with watershed percent land cover, nutrients, and diatom metrics. Land cover correlations used mean axis scores for each site (*n* = 25). Low and high nutrient diatoms were based on OTU relative abundances.

| Variable | NMDS1 | NMDS2 |
|---|---|---|
| % agriculture | -0.76 | 0.18 |
| % urban | 0.60 | -0.26 |
| % forest | 0.70 | -0.14 |
| TP | -0.68 | 0.28 |
| TN | -0.51 | 0.25 |
| $NH_4$ | -0.48 | 0.37 |
| High P diatoms | -0.62 | 0.67 |
| Low P diatoms | 0.78 | -0.5 |
| High N diatoms | -0.49 | 0.72 |
| Low N diatoms | 0.77 | -0.43 |

Table S3. Total phosphorus TITAN results for OTUs with >95% purity and 95% reliability, along with their taxonomy, change points (CP), sample frequencies (Freq), and z-scores. Red and blue cells denote OTUs that increased or decreased, respectively, with increasing TP concentrations. Numbers in parentheses are simply intended to show the bootstrap support between each OTU in our study and the most closely matched sequence in available databases. For statistical analyses, any match <100% similarity was considered a unique OTU (hence some names being reported multiple times, but with different bootstrap support).

| OTU | Taxonomy | CP | Freq | % Freq | z-score | 5% | 10% | 50% | 90% | 95% |
|---|---|---|---|---|---|---|---|---|---|---|
| OTU020 | Bacillariophyceae unclassified (89) | 64.12 | 130 | 46.43 | 6.34 | 53.44 | 61.09 | 69.97 | 152.8 | 190.02 |
| OTU069 | Iconella sp. (84) | 72.16 | 67 | 23.93 | 5.28 | 69.97 | 71.01 | 80.06 | 153.97 | 156.2 |
| OTU078 | Bacillariophyta unclassified (100) | 78.19 | 94 | 33.57 | 5.4 | 70.04 | 72.38 | 80.06 | 123.85 | 133.2 |
| OTU169 | Amphora pediculus (100) | 78.19 | 253 | 90.36 | 7.34 | 55.81 | 69.96 | 84.47 | 137.7 | 138.72 |
| OTU396 | Gomphonema unclassified (76) | 88.63 | 110 | 39.29 | 5.72 | 76.91 | 77.39 | 90.39 | 405.46 | 415.31 |
| OTU025 | Bacillariophyceae unclassified (75) | 89.74 | 139 | 49.64 | 5.62 | 55.88 | 76.49 | 93.66 | 356.36 | 423.07 |
| OTU005 | Bacillariophyta unclassified (91) | 97.87 | 260 | 92.86 | 10.71 | 69.83 | 75.15 | 99.39 | 138.11 | 154.12 |
| OTU074 | Encyonema unclassified (83) | 116.35 | 114 | 40.71 | 6.05 | 50.79 | 80.03 | 118.43 | 136.06 | 148.07 |
| OTU509 | Mayamaea permitis (100) | 116.35 | 95 | 33.93 | 9.79 | 108.95 | 111.2 | 120.82 | 142.18 | 177.27 |
| OTU003 | Diploneis unclassified (82) | 128.22 | 196 | 70 | 11.4 | 113.7 | 118.38 | 126.77 | 138.72 | 143.19 |
| OTU027 | Bacillariophyceae unclassified (86) | 136.06 | 176 | 62.86 | 11.98 | 129.45 | 134.22 | 143.19 | 252.55 | 256.71 |
| OTU024 | Planothidium caputium (100) | 137.7 | 229 | 81.79 | 11.38 | 72.16 | 81.6 | 136.06 | 143.19 | 152.95 |
| OTU001 | Amphora pediculus (99) | 138.72 | 280 | 100 | 4.57 | 29 | 32.54 | 55.23 | 139.52 | 157.17 |
| OTU090 | Halamphora unclassified (92) | 139.29 | 41 | 14.64 | 6.2 | 85.75 | 123.08 | 139.59 | 169.43 | 188.67 |
| OTU019 | Nitzschia soratensis (100) | 141.39 | 134 | 47.86 | 12.61 | 129.44 | 130.12 | 141.39 | 239.07 | 264.2 |
| OTU179 | Mayamaea permitis (81) | 147.68 | 28 | 10 | 5.4 | 102.13 | 116.35 | 151.77 | 214.45 | 216.01 |
| OTU011 | Naviculales unclassified (100) | 151.77 | 221 | 78.93 | 7.29 | 133.2 | 136.06 | 141.39 | 170.87 | 180.33 |
| OTU046 | Bacillariophyceae unclassified (82) | 151.77 | 39 | 13.93 | 6.59 | 128.18 | 134.22 | 152.8 | 362.6 | 557.52 |
| OTU097 | Bacillariophyta unclassified (100) | 151.77 | 53 | 18.93 | 7.48 | 122.49 | 137.48 | 151.77 | 210.85 | 245.44 |
| OTU110 | Nitzschia sigma (84) | 163.94 | 47 | 16.79 | 8.72 | 154.83 | 155.69 | 170.66 | 189.42 | 210 |

| | | | | | | | | | | |
|---|---|---|---|---|---|---|---|---|---|---|
| OTU052 | Bacillariophyceae unclassified (87) | 170.87 | 78 | 27.86 | 6.14 | 118.81 | 124.23 | 213.66 | 456.74 | 463.8 |
| OTU032 | Bacillariophyceae unclassified (85) | 176.88 | 33 | 11.79 | 9.49 | 163.94 | 167.28 | 176.88 | 305.71 | 334.98 |
| OTU368 | Naviculales unclassified (88) | 176.88 | 93 | 33.21 | 8.18 | 120.5 | 136.06 | 171.48 | 210.69 | 212.69 |
| OTU093 | Bacillariophyceae unclassified (92) | 208.89 | 52 | 18.57 | 8.04 | 123.08 | 123.47 | 182.75 | 210.85 | 219.52 |
| OTU140 | Sellaphora seminulum (100) | 210.69 | 21 | 7.5 | 7.32 | 205.57 | 206.8 | 252.43 | 274.3 | 307.17 |
| OTU130 | Chromista unclassified (77) | 213.29 | 32 | 11.43 | 6 | 112.34 | 113.62 | 245.5 | 422.36 | 477.51 |
| OTU082 | Navicula trivialis (99) | 232.7 | 63 | 22.5 | 6.58 | 115.24 | 121.52 | 264.62 | 424.98 | 427.5 |
| OTU404 | Gomphonema unclassified (100) | 276.44 | 52 | 18.57 | 8.97 | 274.3 | 276.26 | 299.07 | 397.01 | 415.3 |
| OTU041 | Gomphonema capitatum (100) | 290.12 | 142 | 50.71 | 4.51 | 56.57 | 62.85 | 276.3 | 397.01 | 402.36 |
| OTU009 | Bacillariophyta unclassified (95) | 299.07 | 79 | 28.21 | 11.76 | 176.1 | 177.03 | 275.09 | 332.02 | 349.55 |
| OTU105 | Gomphonema unclassified (94) | 299.07 | 39 | 13.93 | 7.58 | 216.54 | 269.23 | 307.94 | 423.5 | 427.5 |
| OTU055 | Gomphonema bourbonense (100) | 307.92 | 73 | 26.07 | 5.44 | 90.06 | 275.77 | 307.92 | 650.25 | 650.25 |
| OTU336 | Bacillariophyceae unclassified (75) | 307.92 | 44 | 15.71 | 9.65 | 247.28 | 275.79 | 335.14 | 415.31 | 517.06 |
| OTU067 | Bacillariophyta unclassified (95) | 354.41 | 81 | 28.93 | 13.39 | 213.66 | 216.31 | 284.09 | 354.03 | 354.99 |
| OTU065 | Mayamaea permitis (100) | 355.38 | 151 | 53.93 | 3.9 | 37.15 | 56.8 | 355.38 | 426.82 | 483.4 |
| OTU099 | Bacillariophyta unclassified (95) | 355.38 | 38 | 13.57 | 3.77 | 56.8 | 75.6 | 354.8 | 402.38 | 424.11 |
| OTU088 | Rhoicosphenia abbreviata (92) | 395.5 | 29 | 10.36 | 5.6 | 111.18 | 139.13 | 394.79 | 512.95 | 819.17 |
| OTU028 | Bacillariophyta unclassified (86) | 397.01 | 91 | 32.5 | 11.6 | 290.12 | 294.63 | 318.82 | 402.13 | 408.53 |
| OTU034 | Eukaryota unclassified (100) | 397.01 | 30 | 10.71 | 11.78 | 392.2 | 395.5 | 408.53 | 513.59 | 513.59 |
| OTU079 | Bacillariophyta unclassified (99) | 397.01 | 70 | 25 | 14.54 | 373.55 | 392.61 | 397.01 | 410.58 | 415.31 |
| OTU083 | Bacillariophyta unclassified (92) | 397.01 | 44 | 15.71 | 10.92 | 290.12 | 294.93 | 392.91 | 520.19 | 727.25 |
| OTU054 | Bacillariophyta unclassified (94) | 408.53 | 109 | 38.93 | 4.32 | 57.87 | 100.52 | 365.03 | 417.23 | 422.36 |
| OTU114 | Bacillariophyta unclassified (97) | 411.87 | 12 | 4.29 | 13.73 | 396.59 | 397.01 | 411.87 | 477.51 | 488.64 |
| OTU142 | Bacillariophyta unclassified (81) | 415.31 | 13 | 4.64 | 6.5 | 102.13 | 307.9 | 415.3 | 727.25 | 727.25 |
| OTU094 | Bacillariophyceae unclassified (93) | 442.51 | 31 | 11.07 | 8.74 | 180.94 | 208.02 | 411.87 | 477.51 | 488.64 |
| OTU042 | Naviculales unclassified (80) | 477.51 | 20 | 7.14 | 12.72 | 213.66 | 245.44 | 477.51 | 490.72 | 492.04 |
| OTU634 | Bacillariophyceae unclassified (91) | 477.51 | 20 | 7.14 | 11.02 | 245.55 | 365.03 | 414.61 | 490.72 | 495.45 |
| OTU045 | Bacillariophyta unclassified (99) | 727.25 | 51 | 18.21 | 4.25 | 54.53 | 87.88 | 557 | 727.25 | 800.75 |
| OTU267 | Bacillariophyta unclassified (91) | 727.25 | 37 | 13.21 | 10.84 | 307.94 | 349.55 | 621.22 | 727.25 | 740.75 |
| OTU057 | Bacillariophyta unclassified (100) | 27.42 | 67 | 23.93 | 7.82 | 26.38 | 27.41 | 42.53 | 92.1 | 139.58 |
| OTU075 | Nitzschia fonticola (100) | 37.05 | 75 | 26.79 | 5.43 | 36.25 | 36.35 | 64.22 | 107.86 | 354.41 |

| | | | | | | | | | | |
|---|---|---|---|---|---|---|---|---|---|---|
| OTU059 | Diatoma vulgaris (100) | 37.1 | 7 | 2.5 | 9.99 | 32.3 | 35.1 | 44.95 | 63.03 | 64.12 |
| OTU073 | Cymbella unclassified (93) | 37.1 | 19 | 6.79 | 9.83 | 36.45 | 37.05 | 50.79 | 205.7 | 220.36 |
| OTU068 | Bacillariophyta unclassified (99) | 37.5 | 33 | 11.79 | 5.46 | 32.15 | 32.39 | 58.85 | 205.57 | 207.2 |
| OTU043 | Navicula unclassified (96) | 45.92 | 126 | 45 | 4.36 | 32.15 | 45.56 | 50.39 | 347.11 | 453.06 |
| OTU636 | Sellaphora seminulum (97) | 50.08 | 14 | 5 | 7.22 | 30.75 | 32.65 | 41.2 | 58.98 | 62.5 |
| OTU510 | Nitzschia filiformis (100) | 50.7 | 37 | 13.21 | 13.31 | 32.3 | 34.33 | 53.44 | 100.9 | 109.76 |
| OTU096 | Nitzschia filiformis (81) | 51.74 | 27 | 9.64 | 15.24 | 30.75 | 32.65 | 50.62 | 54.2 | 56.38 |
| OTU033 | Naviculaceae unclassified (94) | 55.23 | 38 | 13.57 | 14.05 | 44.37 | 45.34 | 55.23 | 75.76 | 76.37 |
| OTU076 | Navicula gregaria (99) | 55.81 | 92 | 32.86 | 11.25 | 45.92 | 46.15 | 58.01 | 122.82 | 167.42 |
| OTU112 | Bacillaria paxillifer (100) | 56.8 | 14 | 5 | 6.29 | 36.35 | 37.15 | 59.77 | 164.51 | 168.23 |
| OTU021 | Bacillariophyta unclassified (100) | 58.85 | 42 | 15 | 10.33 | 32.65 | 33.83 | 57.82 | 96.31 | 98.79 |
| OTU106 | Achnanthidium minutissimum (100) | 67.64 | 17 | 6.07 | 7.87 | 36.25 | 37.24 | 65.07 | 102.24 | 107.94 |
| OTU521 | Achnanthidium minutissimum (99) | 67.64 | 19 | 6.79 | 9.83 | 35.85 | 36.2 | 58.98 | 88.43 | 102.57 |
| OTU418 | Navicula rostellata (99) | 69.97 | 116 | 41.43 | 7.96 | 50.47 | 50.79 | 76.5 | 222.34 | 266.75 |
| OTU071 | Amphora ovalis (100) | 73.53 | 174 | 62.14 | 6.14 | 41.2 | 55.81 | 86.78 | 290.12 | 354.81 |
| OTU013 | Nitzschia inconspicua (89) | 78.19 | 70 | 25 | 13.99 | 50.71 | 50.8 | 76.06 | 116.35 | 118.81 |
| OTU061 | Navicula unclassified (91) | 80.06 | 68 | 24.29 | 7.42 | 55.99 | 67.64 | 87.52 | 134.75 | 268.37 |
| OTU072 | Nitzschia inconspicua (100) | 85.75 | 67 | 23.93 | 12.45 | 70.11 | 73.27 | 81.6 | 133.2 | 138.12 |
| OTU036 | Achnanthidium eutrophilum (100) | 87.52 | 109 | 38.93 | 7.87 | 72.16 | 77.85 | 109.91 | 153.97 | 265.35 |
| OTU100 | Karayevia ploenensis var. gessneri (100) | 89.62 | 28 | 10 | 10.14 | 55.23 | 57.64 | 88.4 | 127.23 | 129.44 |
| OTU051 | Cymbellales unclassified (94) | 92.26 | 116 | 41.43 | 6.33 | 58.98 | 60.74 | 98.75 | 133.75 | 272.47 |
| OTU064 | Bacillariaceae unclassified (75) | 97.87 | 84 | 30 | 7.83 | 45.92 | 50.71 | 95.69 | 116.27 | 124.44 |
| OTU010 | Sellaphora minima (93) | 98.9 | 275 | 98.21 | 5.14 | 74.9 | 88.61 | 109.76 | 537.74 | 557.52 |
| OTU087 | Gomphonema pumilum var. rigidum (84 | 102.13 | 56 | 20 | 9.4 | 66.69 | 74.58 | 102.24 | 139.52 | 144.91 |
| OTU048 | Eukaryota unclassified (100) | 102.57 | 45 | 16.07 | 7.06 | 55.43 | 56.8 | 108.02 | 207.2 | 208.89 |
| OTU023 | Cymbella tumida (100) | 110.48 | 178 | 63.57 | 6.18 | 94.43 | 98.79 | 112.61 | 307.9 | 351.97 |
| OTU040 | Stephanodiscaceae unclassified (77) | 112.61 | 16 | 5.71 | 4.25 | 24.49 | 46.12 | 112.34 | 222.34 | 227.51 |
| OTU018 | Craticula unclassified (86) | 118.81 | 173 | 61.79 | 3.48 | 108.78 | 113.62 | 128.22 | 365.03 | 464.36 |
| OTU039 | Nitzschia microcephala (100) | 118.81 | 49 | 17.5 | 10.16 | 36.61 | 36.96 | 116.35 | 138.88 | 153.97 |
| OTU030 | Bacillariophyta unclassified (93) | 121.52 | 185 | 66.07 | 7.99 | 37.4 | 42.23 | 117.37 | 130.12 | 138.33 |
| OTU056 | Nitzschia dissipata (85) | 123.08 | 76 | 27.14 | 8.29 | 57.86 | 67.08 | 109.04 | 264.62 | 269.94 |

| | | | | | | | | | | |
|---|---|---|---|---|---|---|---|---|---|---|
| OTU135 | Fistulifera saprophila (97) | 123.08 | 105 | 37.5 | 12.12 | 99.02 | 107.94 | 139.29 | 188.02 | 198.1 |
| OTU089 | Caloneis sp. (97) | 126.02 | 51 | 18.21 | 5.53 | 88.4 | 102.01 | 127.23 | 212.16 | 215.97 |
| OTU016 | Cymbellaceae unclassified (82) | 129.9 | 118 | 42.14 | 13.13 | 98.79 | 108.89 | 130.12 | 176.74 | 209.59 |
| OTU037 | Bacillariophyta unclassified (97) | 136.06 | 70 | 25 | 7.66 | 80.06 | 115.15 | 136.06 | 179.59 | 182.14 |
| OTU162 | Amphora pediculus (92) | 159.38 | 224 | 80 | 12.35 | 102.57 | 126.92 | 157.17 | 293.88 | 299.07 |
| OTU763 | Bacillariophyceae unclassified (76) | 163.94 | 31 | 11.07 | 7.34 | 44.98 | 79.5 | 127.23 | 166.97 | 170.13 |
| OTU117 | Bacillariophyta unclassified (85) | 169.18 | 20 | 7.14 | 4.95 | 32.15 | 32.3 | 151.77 | 202.8 | 205.7 |
| OTU014 | Bacillariaceae unclassified (96) | 178.01 | 107 | 38.21 | 7.59 | 111.2 | 126.02 | 178.01 | 241.28 | 245.56 |
| OTU084 | Nitzschia filiformis (97) | 222.34 | 81 | 28.93 | 7.55 | 47.8 | 52.5 | 222.34 | 268.17 | 271.26 |
| OTU017 | Thalassiosirales unclassified (84) | 243.27 | 82 | 29.29 | 5.62 | 141.39 | 155.34 | 236.25 | 251.78 | 257.51 |
| OTU437 | Navicula unclassified (91) | 269.71 | 98 | 35 | 3.73 | 46.14 | 62.72 | 170.31 | 290.12 | 389.61 |
| OTU006 | Nitzschia inconspicua (100) | 271.27 | 259 | 92.5 | 11.61 | 214.85 | 232.7 | 275.77 | 359.95 | 363.94 |
| OTU070 | Navicula cryptotenella (100) | 275.79 | 95 | 33.93 | 6.31 | 89.74 | 90.06 | 271.27 | 276.56 | 289.62 |
| OTU029 | Nitzschia unclassified (100) | 285.85 | 177 | 63.21 | 8.29 | 75.14 | 90.38 | 271.26 | 290.49 | 294.93 |
| OTU116 | Nitzschia sigmoidea (100) | 285.85 | 59 | 21.07 | 4.83 | 89.74 | 195.03 | 269.23 | 289.62 | 293.88 |
| OTU053 | Surirella minuta (85) | 286.35 | 114 | 40.71 | 4.25 | 195.46 | 267.62 | 289.99 | 396.61 | 397 |
| OTU202 | Amphora pediculus (85) | 290.12 | 254 | 90.71 | 9.78 | 232.7 | 245.21 | 290.12 | 398.84 | 431.05 |
| OTU022 | Nitzschia bulnheimiana (100) | 336.81 | 214 | 76.43 | 4.62 | 131.89 | 196.21 | 336.44 | 354.03 | 356.45 |
| OTU015 | Nitzschia palea (100) | 381.74 | 247 | 88.21 | 3.48 | 138.5 | 162.25 | 335.14 | 476.53 | 506.2 |

Table S4. Total Nitrogen TITAN results for OTUs with >95% purity and 95% reliability, along with their taxonomy, change points (CP), sample frequencies (Freq), and z-scores. Red and blue cells denote OTUs that increased or decreased, respectively, with increasing TN concentrations. Numbers in parentheses are simply intended to show the bootstrap support between each OTU in our study and the most closely matched sequence in available databases. For statistical analyses, any match <100% similarity was considered a unique OTU (hence some names being reported multiple times, but with different bootstrap support).

| OTU | Taxonomy | CP | Freq | % Freq | z-score | 5% | 10% | 50% | 90% | 95% |
|---|---|---|---|---|---|---|---|---|---|---|
| OTU011 | Naviculales unclassified (100) | 364.5 | 222 | 0.79 | 5.48 | 346 | 353 | 364.5 | 405.5 | 470 |
| OTU169 | Amphora pediculus (100) | 364.5 | 254 | 0.9 | 6.61 | 322 | 353 | 364.5 | 519 | 558.1 |
| OTU044 | Nitzschia acidoclinata (99) | 446.5 | 141 | 0.5 | 4.75 | 353 | 426 | 447.5 | 608.1 | 787.5 |
| OTU105 | Gomphonema unclassified (94) | 503.5 | 39 | 0.14 | 5.33 | 479.5 | 489 | 554.5 | 1820 | 2235 |
| OTU082 | Navicula trivialis (99) | 553 | 63 | 0.22 | 8.75 | 463 | 513.5 | 571 | 787.5 | 811.5 |
| OTU179 | Mayamaea permitis (81) | 561.5 | 28 | 0.1 | 3.47 | 425.5 | 430 | 561.5 | 956.9 | 1820 |
| OTU009 | Bacillariophyta unclassified (95) | 591 | 79 | 0.28 | 7.83 | 560 | 573 | 593.5 | 759.5 | 776 |
| OTU509 | Mayamaea permitis (100) | 602 | 96 | 0.34 | 4.66 | 369.5 | 403 | 574 | 682 | 787.5 |
| OTU027 | Bacillariophyceae unclassified (86) | 620 | 177 | 0.63 | 8.91 | 466 | 472 | 624 | 658 | 784.1 |
| OTU097 | Bacillariophyta unclassified (100) | 624.5 | 54 | 0.19 | 5.92 | 447.5 | 463 | 619 | 728.5 | 822.5 |
| OTU368 | Naviculales unclassified (88) | 624.5 | 93 | 0.33 | 8.7 | 591 | 603.5 | 624.5 | 803 | 814 |
| OTU001 | Amphora pediculus (99) | 654.5 | 281 | 1 | 4.82 | 296.5 | 318.5 | 651 | 1070 | 1225.3 |
| OTU005 | Bacillariophyta unclassified (91) | 665 | 261 | 0.93 | 10.98 | 623.3 | 658 | 686 | 747 | 771.5 |
| OTU067 | Bacillariophyta unclassified (95) | 665 | 81 | 0.29 | 10.66 | 600.9 | 642 | 677 | 728.8 | 855 |
| OTU110 | Nitzschia sigma (84) | 669 | 48 | 0.17 | 6.54 | 539.5 | 572 | 658 | 683 | 684 |
| OTU032 | Bacillariophyceae unclassified (85) | 696.5 | 33 | 0.12 | 6.57 | 507.5 | 509 | 689 | 778.5 | 847.1 |
| OTU054 | Bacillariophyta unclassified (94) | 704.5 | 109 | 0.39 | 8.63 | 704.4 | 708.5 | 895.5 | 1420 | 1575 |
| OTU019 | Nitzschia soratensis (100) | 824.5 | 135 | 0.48 | 8.68 | 613.5 | 659.4 | 814 | 861 | 880 |
| OTU024 | Planothidium caputium (100) | 824.5 | 230 | 0.82 | 7.16 | 675 | 684 | 822.5 | 861 | 871 |
| OTU130 | Chromista unclassified (77) | 824.5 | 32 | 0.11 | 9.04 | 591.1 | 635.4 | 824.5 | 2009 | 2205 |

| | | | | | | | | | | |
|---|---|---|---|---|---|---|---|---|---|---|
| OTU119 | Discostella unclassified (79) | 840.5 | 19 | 0.07 | 5.51 | 822.4 | 825 | 863.5 | 2205 | 2205.5 |
| OTU042 | Naviculales unclassified (80) | 895.5 | 20 | 0.07 | 10.06 | 822.5 | 885 | 898.5 | 2190 | 2205 |
| OTU094 | Bacillariophyceae unclassified (93) | 906.5 | 32 | 0.11 | 8.53 | 683.5 | 721 | 905.5 | 2205 | 2350.5 |
| OTU065 | Mayamaea permitis (100) | 1162.5 | 152 | 0.54 | 5.83 | 574 | 678 | 886 | 1316 | 1765 |
| OTU066 | Bacillariophyta unclassified (98) | 1230 | 85 | 0.3 | 6.64 | 787.5 | 876 | 1207.5 | 1740 | 1820 |
| OTU079 | Bacillariophyta unclassified (99) | 1820 | 70 | 0.25 | 4 | 455 | 460.5 | 1600 | 1847.5 | 2065 |
| OTU088 | Rhoicosphenia abbreviata (92) | 1820 | 29 | 0.1 | 5.61 | 568.5 | 591 | 1790 | 2568.5 | 2568.5 |
| OTU114 | Bacillariophyta unclassified (97) | 1820 | 12 | 0.04 | 15.97 | 1067 | 1620 | 1805 | 2045.5 | 2065 |
| OTU052 | Bacillariophyceae unclassified (87) | 2045 | 78 | 0.28 | 8.79 | 796.5 | 811.5 | 1878.8 | 2065 | 2130 |
| OTU634 | Bacillariophyceae unclassified (91) | 2065 | 20 | 0.07 | 13.18 | 919 | 924.5 | 1790 | 2065 | 2225.5 |
| OTU085 | Cocconeis pediculus (100) | 206 | 16 | 0.06 | 14.31 | 178.5 | 178.5 | 205 | 293.5 | 299 |
| OTU033 | Naviculaceae unclassified (94) | 228.5 | 38 | 0.14 | 7.46 | 169 | 209.5 | 289 | 681.5 | 738 |
| OTU100 | Karayevia ploenensis var. gessneri (100) | 228.5 | 28 | 0.1 | 7.29 | 173 | 173 | 228.5 | 572.1 | 759.5 |
| OTU211 | Eukaryota unclassified (100) | 228.5 | 20 | 0.07 | 6.79 | 169 | 173 | 221.3 | 546.5 | 561.6 |
| OTU116 | Nitzschia sigmoidea (100) | 247.5 | 60 | 0.21 | 7.4 | 209.5 | 221 | 247.5 | 426 | 713 |
| OTU073 | Cymbella unclassified (93) | 263 | 19 | 0.07 | 14.27 | 192.8 | 210.5 | 263 | 276.1 | 297.5 |
| OTU510 | Nitzschia filiformis (100) | 271 | 37 | 0.13 | 6.06 | 183.9 | 213.5 | 433 | 666.5 | 668 |
| OTU061 | Navicula unclassified (91) | 280.5 | 68 | 0.24 | 4.97 | 178.5 | 178.5 | 258.3 | 1105 | 1200 |
| OTU059 | Diatoma vulgaris (100) | 299 | 7 | 0.02 | 11.3 | 189.5 | 201 | 299 | 323 | 405 |
| OTU048 | Eukaryota unclassified (100) | 305 | 45 | 0.16 | 13.94 | 222 | 223 | 307 | 459.5 | 463 |
| OTU036 | Achnanthidium eutrophilum (100) | 315 | 109 | 0.39 | 12.92 | 305 | 312 | 332 | 369.5 | 401.5 |
| OTU075 | Nitzschia fonticola (100) | 331 | 75 | 0.27 | 6.19 | 319 | 325.5 | 367 | 675 | 679.5 |
| OTU087 | Gomphonema pumilum var. rigidum (84) | 332.5 | 56 | 0.2 | 7.73 | 279 | 283 | 334.5 | 362.1 | 425.7 |
| OTU013 | Nitzschia inconspicua (89) | 335 | 70 | 0.25 | 9.53 | 287 | 293.5 | 335 | 629.7 | 660.5 |
| OTU112 | Bacillaria paxillifer (100) | 335 | 14 | 0.05 | 10.7 | 178.5 | 213.2 | 299 | 337 | 351 |
| OTU076 | Navicula gregaria (99) | 343 | 93 | 0.33 | 6.58 | 263 | 272 | 345 | 683 | 728.5 |
| OTU016 | Cymbellaceae unclassified (82) | 353.5 | 118 | 0.42 | 11.38 | 333 | 337 | 358.5 | 655.1 | 667 |
| OTU072 | Nitzschia inconspicua (100) | 354.5 | 67 | 0.24 | 7.15 | 296.5 | 322.8 | 361.5 | 825 | 836 |
| OTU010 | Sellaphora minima (93) | 364.5 | 276 | 0.98 | 4.52 | 335 | 355.5 | 461.5 | 1230 | 1255 |
| OTU006 | Nitzschia inconspicua (100) | 376 | 260 | 0.93 | 4.46 | 333 | 369.5 | 663.5 | 1280 | 1290 |
| OTU037 | Bacillariophyta unclassified (97) | 377.5 | 70 | 0.25 | 4.63 | 178.5 | 282.9 | 395 | 836.1 | 852 |

| | | | | | | | | | | |
|---|---|---|---|---|---|---|---|---|---|---|
| OTU039 | Nitzschia microcephala (100) | 403 | 49 | 0.17 | 10.62 | 287 | 299 | 403 | 612 | 614.5 |
| OTU117 | Bacillariophyta unclassified (85) | 405.5 | 20 | 0.07 | 5.99 | 271 | 276.5 | 376 | 614.5 | 617 |
| OTU026 | Bacillariophyta unclassified (99) | 421.5 | 186 | 0.66 | 6.6 | 315 | 373.5 | 434 | 539.5 | 606 |
| OTU021 | Bacillariophyta unclassified (100) | 440 | 42 | 0.15 | 6.6 | 206 | 210.5 | 398 | 507.5 | 524 |
| OTU018 | Craticula unclassified (86) | 462 | 174 | 0.62 | 3.19 | 326.5 | 373.5 | 480 | 829.5 | 983.4 |
| OTU109 | Bacillariophyta unclassified (98) | 463 | 48 | 0.17 | 7.85 | 178.5 | 220.5 | 464 | 519 | 519 |
| OTU030 | Bacillariophyta unclassified (93) | 466 | 185 | 0.66 | 8.39 | 430 | 434 | 466 | 492.5 | 506 |
| OTU096 | Nitzschia filiformis (81) | 467 | 27 | 0.1 | 6.98 | 210.5 | 213.5 | 492.5 | 574.7 | 610.5 |
| OTU014 | Bacillariaceae unclassified (96) | 476 | 107 | 0.38 | 6.09 | 341 | 394 | 476 | 560 | 569.5 |
| OTU071 | Amphora ovalis (100) | 485.5 | 174 | 0.62 | 5.51 | 446.5 | 457 | 567.3 | 798 | 860.7 |
| OTU007 | Melosira varians (100) | 505 | 232 | 0.83 | 3.67 | 418 | 430 | 501.5 | 780 | 797.9 |
| OTU045 | Bacillariophyta unclassified (99) | 541.5 | 51 | 0.18 | 6.51 | 173 | 222 | 541 | 553 | 604.5 |
| OTU267 | Bacillariophyta unclassified (91) | 548 | 37 | 0.13 | 3.98 | 127 | 134.5 | 543.5 | 552.5 | 560 |
| OTU202 | Amphora pediculus (85) | 602 | 255 | 0.91 | 4.43 | 455 | 507.5 | 637 | 1255 | 1265 |
| OTU106 | Achnanthidium minutissimum (100) | 607 | 17 | 0.06 | 3.98 | 326.5 | 331.5 | 590 | 665 | 669 |
| OTU162 | Amphora pediculus (92) | 614.5 | 225 | 0.8 | 6.84 | 446.5 | 455 | 644.8 | 914.5 | 919 |
| OTU064 | Bacillariaceae unclassified (75) | 635.5 | 85 | 0.3 | 5.61 | 452.5 | 604.5 | 639 | 780.5 | 782.5 |
| OTU062 | Halamphora montana (99) | 662.5 | 127 | 0.45 | 5.67 | 549.5 | 581.5 | 662.5 | 676 | 811.5 |
| OTU135 | Fistulifera saprophila (97) | 665 | 105 | 0.37 | 6.78 | 332.5 | 371 | 660.5 | 774 | 781.1 |
| OTU280 | Amphora pediculus (96) | 666.5 | 169 | 0.6 | 4.56 | 295.5 | 335 | 664 | 731.6 | 781.1 |
| OTU056 | Nitzschia dissipata (85) | 668 | 77 | 0.27 | 5.91 | 331.5 | 442.4 | 616.5 | 727.5 | 736 |
| OTU521 | Achnanthidium minutissimum (99) | 668 | 19 | 0.07 | 4.87 | 304 | 355 | 665 | 669 | 674.1 |
| OTU029 | Nitzschia unclassified (100) | 727.5 | 178 | 0.63 | 5.62 | 272 | 275 | 665 | 848 | 873.5 |
| OTU023 | Cymbella tumida (100) | 737 | 179 | 0.64 | 5.34 | 616 | 665.5 | 729 | 930 | 1010 |
| OTU084 | Nitzschia filiformis (97) | 773 | 82 | 0.29 | 6.08 | 725.3 | 733.5 | 772 | 814 | 821.1 |
| OTU017 | Thalassiosirales unclassified (84) | 775 | 82 | 0.29 | 3.37 | 178.5 | 415.5 | 730.5 | 777.5 | 780.5 |
| OTU022 | Nitzschia bulnheimiana (100) | 1130 | 215 | 0.77 | 4.38 | 618.9 | 821 | 1110 | 1310 | 1325 |
| OTU060 | Nitzschia sp. (100) | 1215 | 80 | 0.28 | 3.11 | 173 | 189.5 | 796.5 | 1220 | 1230 |
| OTU008 | Nitzschia amphibia (100) | 1345 | 262 | 0.93 | 4.16 | 396.4 | 404 | 1045 | 1420 | 1440 |

Table S5. OTUs from random forest models having $R^2 > 0$ (i.e., variation explained using relative abundances). Parentheses show the bootstrap support between each OTU in our study and the most closely matched sequence in available databases. Numbers in parentheses the bootstrap support between each OTU in our study and the most closely matched sequence in available databases. For statistical analyses, any match <100% similarity was considered a unique OTU (hence some names being reported multiple times, but with different bootstrap support).

| OTU | Taxonomy | $R^2$ |
|---|---|---|
| OTU009 | Bacillariophyta unclassified (95) | 0.614 |
| OTU032 | Bacillariophyceae unclassified (85) | 0.478 |
| OTU106 | Achnanthidium minutissimum (100) | 0.474 |
| OTU021 | Bacillariophyta unclassified (100) | 0.417 |
| OTU636 | Sellaphora seminulum (97) | 0.382 |
| OTU003 | Diploneis unclassified (82) | 0.381 |
| OTU039 | Nitzschia microcephala (100) | 0.378 |
| OTU084 | Nitzschia filiformis (97) | 0.342 |
| OTU169 | Amphora pediculus (100) | 0.340 |
| OTU018 | Craticula unclassified (86) | 0.311 |
| OTU014 | Bacillariaceae unclassified (96) | 0.308 |
| OTU020 | Bacillariophyceae unclassified (89) | 0.295 |
| OTU005 | Bacillariophyta unclassified (91) | 0.293 |
| OTU024 | Planothidium caputium (100) | 0.262 |
| OTU509 | Mayamaea permitis (100) | 0.252 |
| OTU280 | Amphora pediculus (96) | 0.244 |
| OTU041 | Gomphonema capitatum (100) | 0.240 |
| OTU008 | Nitzschia amphibia (100) | 0.238 |
| OTU202 | Amphora pediculus (85) | 0.234 |
| OTU404 | Gomphonema unclassified (100) | 0.221 |
| OTU046 | Bacillariophyceae unclassified (82) | 0.217 |
| OTU368 | Naviculales unclassified (88) | 0.209 |
| OTU074 | Encyonema unclassified (83) | 0.205 |
| OTU072 | Nitzschia inconspicua (100) | 0.188 |
| OTU093 | Bacillariophyceae unclassified (92) | 0.188 |
| OTU037 | Bacillariophyta unclassified (97) | 0.183 |
| OTU028 | Bacillariophyta unclassified (86) | 0.181 |

| | | |
|---|---|---|
| OTU079 | Bacillariophyta unclassified (99) | 0.178 |
| OTU010 | Sellaphora minima (93) | 0.175 |
| OTU075 | Nitzschia fonticola (100) | 0.174 |
| OTU110 | Nitzschia sigma (84) | 0.172 |
| OTU001 | Amphora pediculus (99) | 0.171 |
| OTU029 | Nitzschia unclassified (100) | 0.167 |
| OTU056 | Nitzschia dissipata (85) | 0.165 |
| OTU038 | Navicula unclassified (99) | 0.165 |
| OTU013 | Nitzschia inconspicua (89) | 0.158 |
| OTU011 | Naviculales unclassified (100) | 0.150 |
| OTU071 | Amphora ovalis (100) | 0.149 |
| OTU064 | Bacillariaceae unclassified (75) | 0.144 |
| OTU051 | Cymbellales unclassified (94) | 0.139 |
| OTU055 | Gomphonema bourbonense (100) | 0.130 |
| OTU067 | Bacillariophyta unclassified (95) | 0.128 |
| OTU162 | Amphora pediculus (92) | 0.128 |
| OTU076 | Navicula gregaria (99) | 0.127 |
| OTU140 | Sellaphora seminulum (100) | 0.126 |
| OTU090 | Halamphora unclassified (92) | 0.125 |
| OTU078 | Bacillariophyta unclassified (100) | 0.123 |
| OTU179 | Mayamaea permitis (81) | 0.122 |
| OTU060 | Nitzschia sp. (100) | 0.121 |
| OTU115 | Bacillariophyceae unclassified (96) | 0.121 |
| OTU100 | Karayevia ploenensis var. gessneri (100) | 0.119 |
| OTU083 | Bacillariophyta unclassified (92) | 0.108 |
| OTU004 | Sellaphora minima (100) | 0.104 |
| OTU070 | Navicula cryptotenella (100) | 0.103 |
| OTU891 | Nitzschia unclassified (96) | 0.102 |
| OTU082 | Navicula trivialis (99) | 0.100 |
| OTU034 | Eukaryota unclassified (100) | 0.099 |
| OTU396 | Gomphonema unclassified (76) | 0.098 |
| OTU033 | Naviculaceae unclassified (94) | 0.095 |
| OTU027 | Bacillariophyceae unclassified (86) | 0.091 |
| OTU007 | Melosira varians (100) | 0.091 |
| OTU036 | Achnanthidium eutrophilum (100) | 0.088 |
| OTU045 | Bacillariophyta unclassified (99) | 0.085 |
| OTU087 | Gomphonema pumilum var. rigidum (84) | 0.085 |
| OTU026 | Bacillariophyta unclassified (99) | 0.082 |
| OTU109 | Bacillariophyta unclassified (98) | 0.077 |
| OTU065 | Mayamaea permitis (100) | 0.077 |
| OTU088 | Rhoicosphenia abbreviata (92) | 0.075 |

| | | |
|---|---|---|
| OTU006 | Nitzschia inconspicua (100) | 0.071 |
| OTU267 | Bacillariophyta unclassified (91) | 0.071 |
| OTU016 | Cymbellaceae unclassified (82) | 0.068 |
| OTU135 | Fistulifera saprophila (97) | 0.065 |
| OTU097 | Bacillariophyta unclassified (100) | 0.064 |
| OTU069 | Iconella sp. (84) | 0.060 |
| OTU023 | Cymbella tumida (100) | 0.053 |
| OTU092 | Navicula veneta (100) | 0.046 |
| OTU418 | Navicula rostellata (99) | 0.045 |
| OTU336 | Bacillariophyceae unclassified (75) | 0.043 |
| OTU118 | Epithemia sorex (99) | 0.038 |
| OTU053 | Surirella minuta (85) | 0.034 |
| OTU058 | Bacillariophyceae unclassified (78) | 0.032 |
| OTU050 | Bacillariophyta unclassified (90) | 0.032 |
| OTU061 | Navicula unclassified (91) | 0.032 |
| OTU017 | Thalassiosirales unclassified (84) | 0.032 |
| OTU116 | Nitzschia sigmoidea (100) | 0.030 |
| OTU040 | Stephanodiscaceae unclassified (77) | 0.028 |
| OTU066 | Bacillariophyta unclassified (98) | 0.026 |
| OTU142 | Bacillariophyta unclassified (81) | 0.019 |
| OTU062 | Halamphora montana (99) | 0.018 |
| OTU510 | Nitzschia filiformis (100) | 0.018 |
| OTU054 | Bacillariophyta unclassified (94) | 0.018 |
| OTU453 | Gomphonema unclassified (94) | 0.017 |
| OTU105 | Gomphonema unclassified (94) | 0.014 |
| OTU063 | Bacillariophyta unclassified (97) | 0.012 |
| OTU211 | Eukaryota unclassified (100) | 0.011 |
| OTU130 | Chromista unclassified (77) | 0.010 |
| OTU112 | Bacillaria paxillifer (100) | 0.007 |
| OTU002 | Cyclotella meneghiniana (96) | 0.006 |
| OTU437 | Navicula unclassified (91) | 0.002 |

Table S6. Summary of diatom responses to total phosphorus (TP). Values coincide with those plotted in figure 6 and are ordered from lowest to highest mid-response. BRT = boosted regression tree results in which values represent regions of partial dependence plots within which greatest rates of change occurred. TITAN = threshold indicator taxa analysis results including change points (CP) and points at which steepest portions of the cumulative frequency plot of bootstrapped change points existed. GF = gradient forest results in which mid-response values were peaks in the split density plot with the starts and ends of responses spanning the increase and decrease of splits surrounding peaks with ratios > 1.

| TP responses | Mid-response (µg/L) | Start of response (µg/L) | End of response (µg/L) |
|---|---|---|---|
| LP sumZ | 98 | 60 | 135 |
| LP CP[1] | 96 | 68 | 141 |
| HP sumZ1 | 148 | 135 | 160 |
| HP CP[1] | 152 | 139 | 310 |
| HP sumZ2 | 215 | 210 | 220 |
| GF1 | 38 | 20 | 75 |
| GF2 | 107 | 75 | 134 |
| GF3 | 295 | 250 | 367 |
| GF4 | 418 | 405 | 430 |
| GF5 | 513 | 500 | 525 |
| GF6 | 722 | 711 | 733 |
| NMDS1 BRT1 | 107 | 28 | 185 |
| NMDS1 BRT2 | 296 | 283 | 308 |
| LP BRT1 | 40 | 32 | 48 |
| LP BRT2 | 77 | 74 | 80 |
| LP BRT3 | 128 | 118 | 138 |
| LP BRT4 | 250 | 207 | 293 |
| HP BRT1 | 52.5 | 25 | 80 |
| HP BRT2 | 152 | 129 | 175 |
| HP BRT3 | 287 | 283 | 290 |

[1]Mid-response is the identified change point with the $5^{th}$ and $95^{th}$ percentiles being the start and end of the response based on bootstrapped change points in TITAN.

Table S7. Summary of diatom responses to total nitrogen (TN). Values coincide with those plotted in figure 6 and are ordered from lowest to highest mid-response. BRT = boosted regression tree results in which values represent regions of partial dependence plots within which greatest rates of change occurred. TITAN = threshold indicator taxa analysis results including change points (CP) and points at which steepest portions of the cumulative frequency plot of bootstrapped change points existed. GF = gradient forest results in which mid-response values were peaks in the split density plot with the starts and ends of responses spanning the increase and decrease of splits surrounding peaks with ratios > 1.

| TN responses | Mid-response (µg/L) | Start of response (µg/L) | End of response (µg/L) |
|---|---|---|---|
| LN CP[1] | 333 | 307 | 667 |
| LN sumZ1 | 350 | 250 | 450 |
| LN sumZ2 | 605 | 560 | 650 |
| HN sumZ | 715 | 560 | 870 |
| HN CP[1] | 823 | 574 | 851 |
| GF1 | 493 | 153 | 833 |
| GF2 | 4362 | 4188 | 4558 |
| GF3 | 4875 | 4660 | 5070 |
| NMDS1 BRT | 559 | 280 | 838 |
| LN BRT1 | 406 | 281 | 531 |
| LN BRT2 | 691 | 594 | 788 |
| HN BRT1 | 694 | 538 | 850 |
| HNBRT2 | 1278 | 1212 | 1344 |

[1]Mid-response is the identified change point with the 5$^{th}$ and 95$^{th}$ percentiles being the start and end of the response based on bootstrapped change points in TITAN.

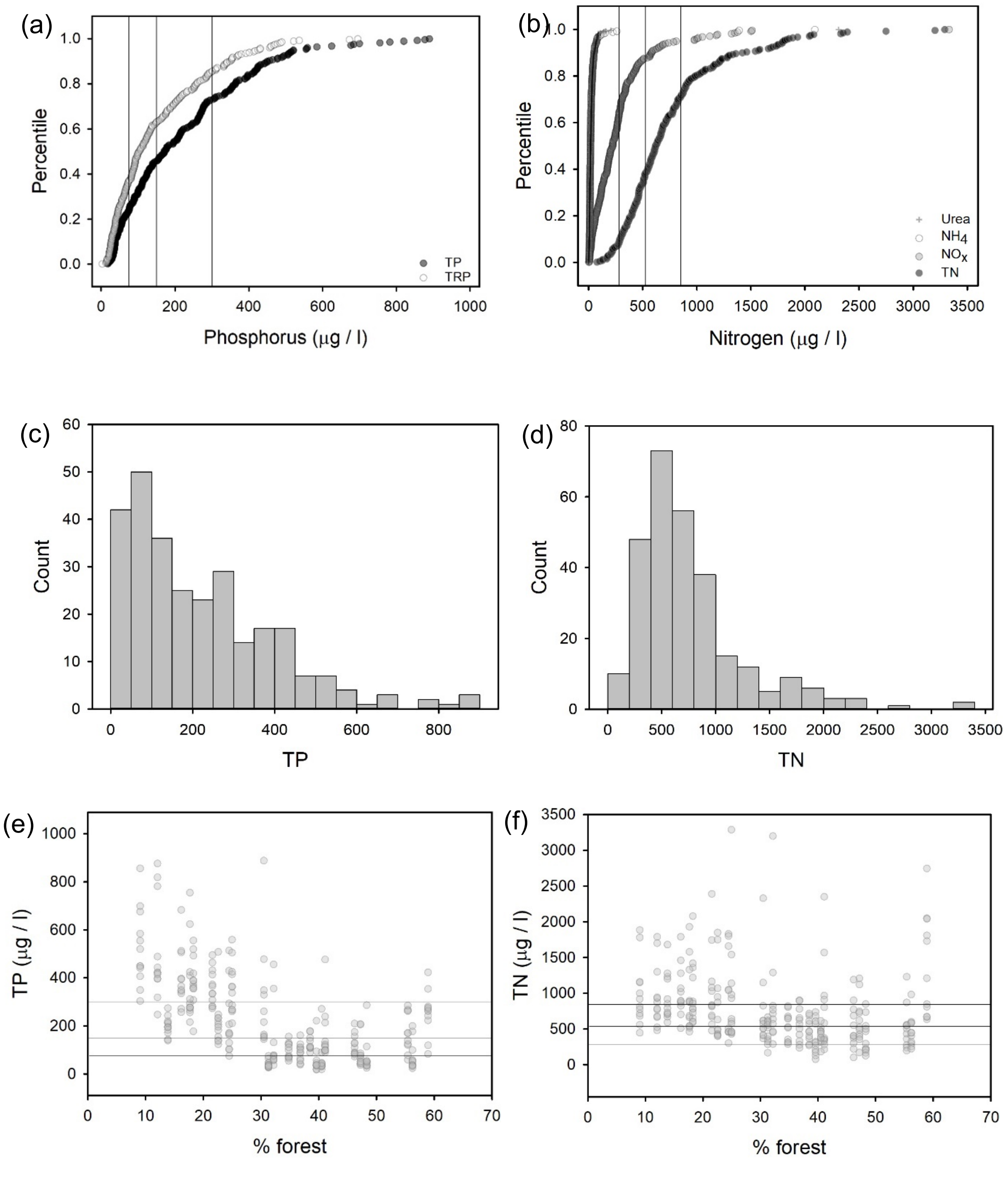


Figure S1. Distribution of nutrient data collected from all weeks (*n* = 280 with one TN outlier of 6560 μg/L removed). TP (a) and TN (b) concentrations showing evenly distributed and continuous gradients. Histograms to further aid with interpreting data distributions (c, d). Within site variation in nutrient concentrations (e, f). Vertical lines in (a) and (b) and horizontal lines in

(e) and (f) demarcate portions of TP and TN gradients within which numerous and large changes in diatom assemblages occurred as reported in the manuscript and as summarized in figure 6. Collectively, these figures show the value of including all nutrient data for statistics used to examine diatom relationships with TP and TN. Each vertical distribution of points (samples over time) is an individual site identified by its watershed percent forest.

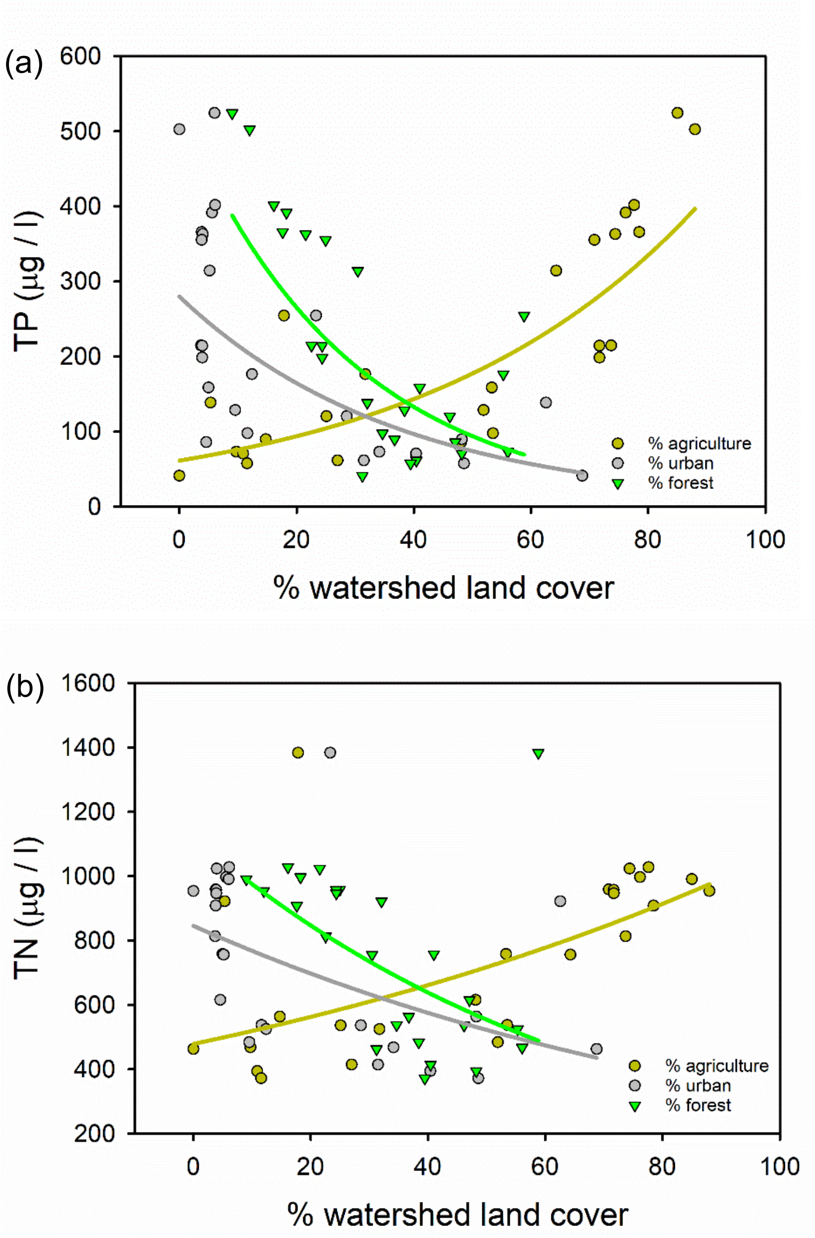


Figure S2. Relationships between watershed land cover and site means of TP (a) and TN (b). For TP: log TP = 0.0092 × % agriculture + 1.789 ($R^2$ = 0.69), = -0.012 × % urban + 2.447 ($R^2$ = 0.53), = -0.015 × % forest + 2.724 ($R^2$ = 0.42). For TN: log TN = 0.0035 × % agriculture + 2.680 ($R^2$ = 0.40), = -0.0042 × % urban + 2.927 ($R^2$ = 0.28), = -0.0061 × % forest + 3.05 ($R^2$ = 0.28). When excluding the TN outlier of 1383 µg/L, % agriculture $R^2$ = 0.62, % urban $R^2$ = 0.35, and % forest $R^2$ = 0.63.

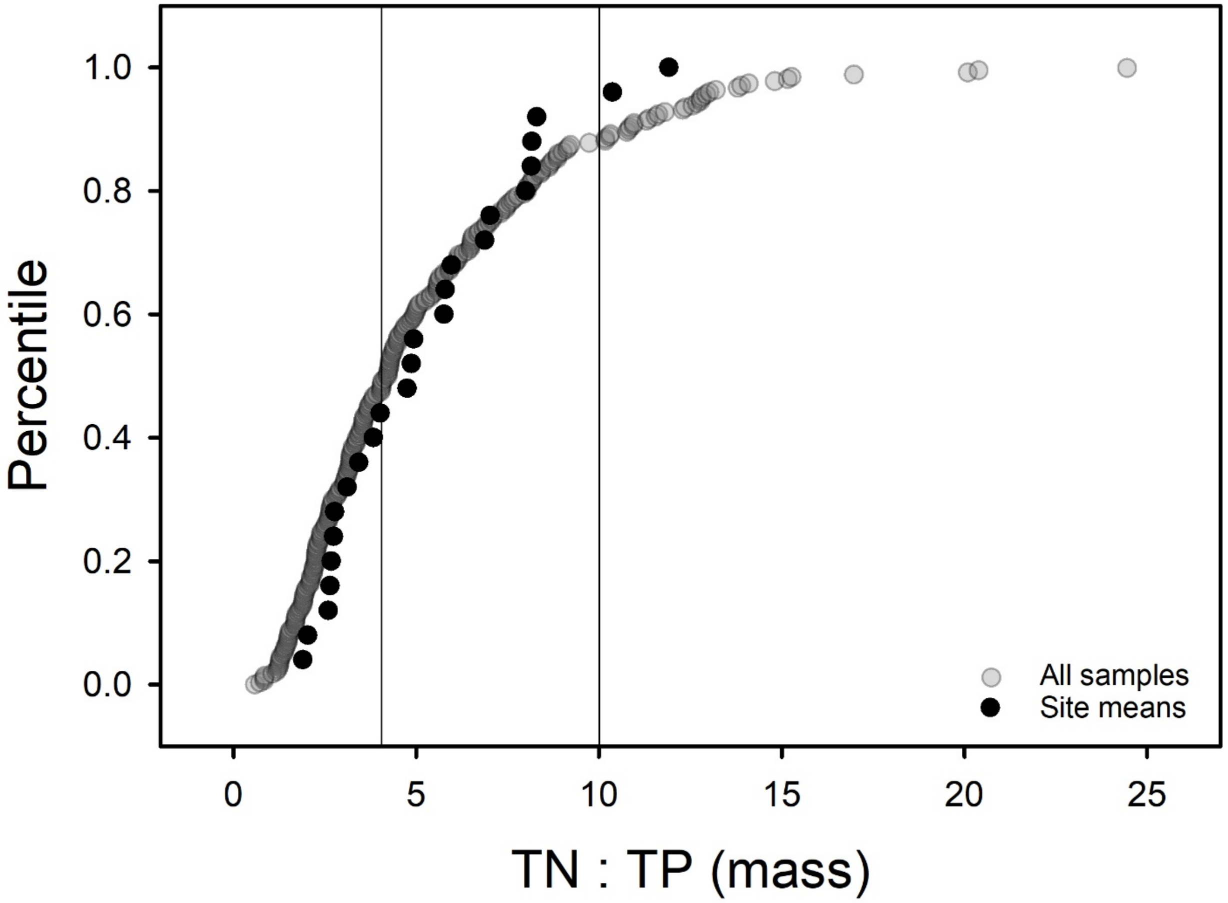


Figure S3. TN:TP mass ratios for all samples. Vertical lines highlight ratios often interpreted as indicating possible N limitation (< 4; 133 samples [47%]), co-limitation or no limitation (4–10; 114 samples [41%]), and P limitation (> 10; 34 samples [12%]). Of 25 sites, 10 had mean ratios < 4, 13 had mean ratios of 4–10, and 2 had mean ratios > 10.

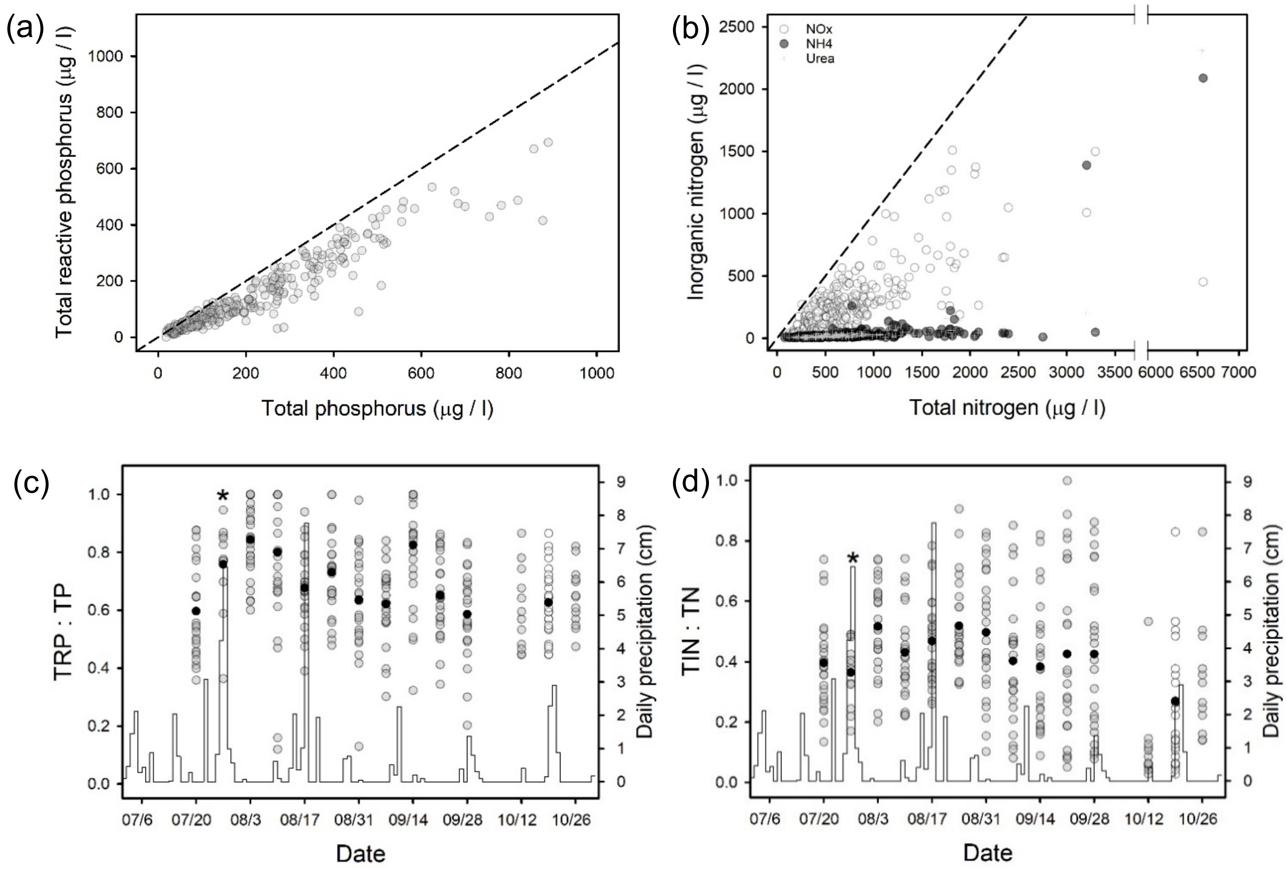


Figure S4. Total reactive phosphorus typically comprised the majority of TP (a) and $NO_3$-$NO_2$ ($NO_x$) was the most abundant form of inorganic nitrogen at sites (b). Total reactive phosphorus to total phosphorus ratios (TRP:TP) were variable over time with weekly means ranging from 0.586–0.844 (c). Total inorganic nitrogen to total nitrogen ratios (TIN:TN) also varied over time and were mostly between 0.364–0.59 (d). Black circles in (c) and (d) show weekly means of all sites. Only 12 sites were sampled the week of 7/23 (asterisks) and these tended to be higher nutrient sites. For visual comparisons and examining means of all sites, white circles for 10/19 combine data from 10/12 (12 sites) and 10/26 (13 sites) because sampling logistics limited the number of sites sampled each of those weeks.

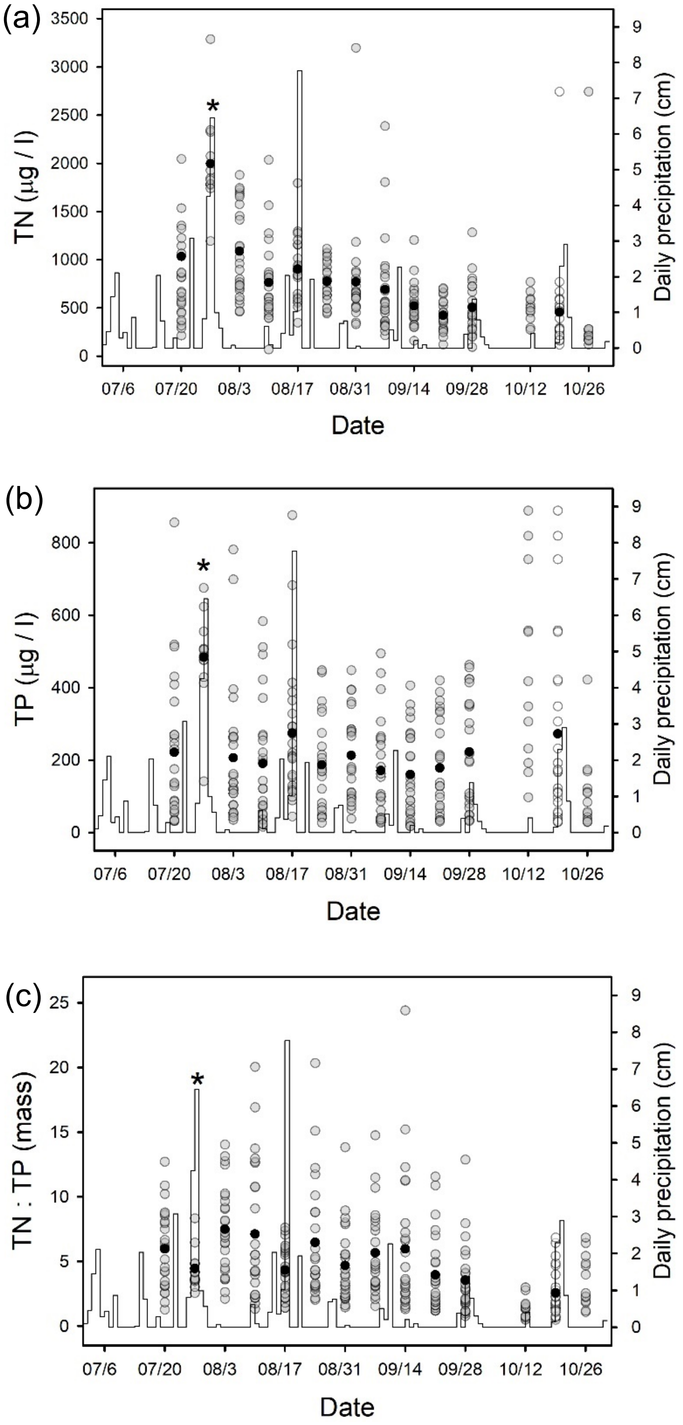


Figure S5. Variability in TN (a), TP (b), TN:TP (c), and daily precipitation over time. Black circles show weekly means of all sites. Only 12 sites were sampled the week of 7/23 (asterisks) and these tended to be higher nutrient sites. These sites did increase in TP and TN concentrations

for one week relative to their mean concentrations in the previous and following weeks likely due to the precipitation event (7/20 TP = 365, TN = 1518; 7/27 TP = 484, TN = 1997, 8/3 TP = 324, TN = 1452). The second major precipitation event around 8/16 only created a minor increase in TP and TN. For visual comparisons and examining means of all sites, white circles for 10/19 combine data from 10/12 (12 sites) and 10/26 (13 sites) because sampling logistics limited the number of sites sampled each of those weeks.

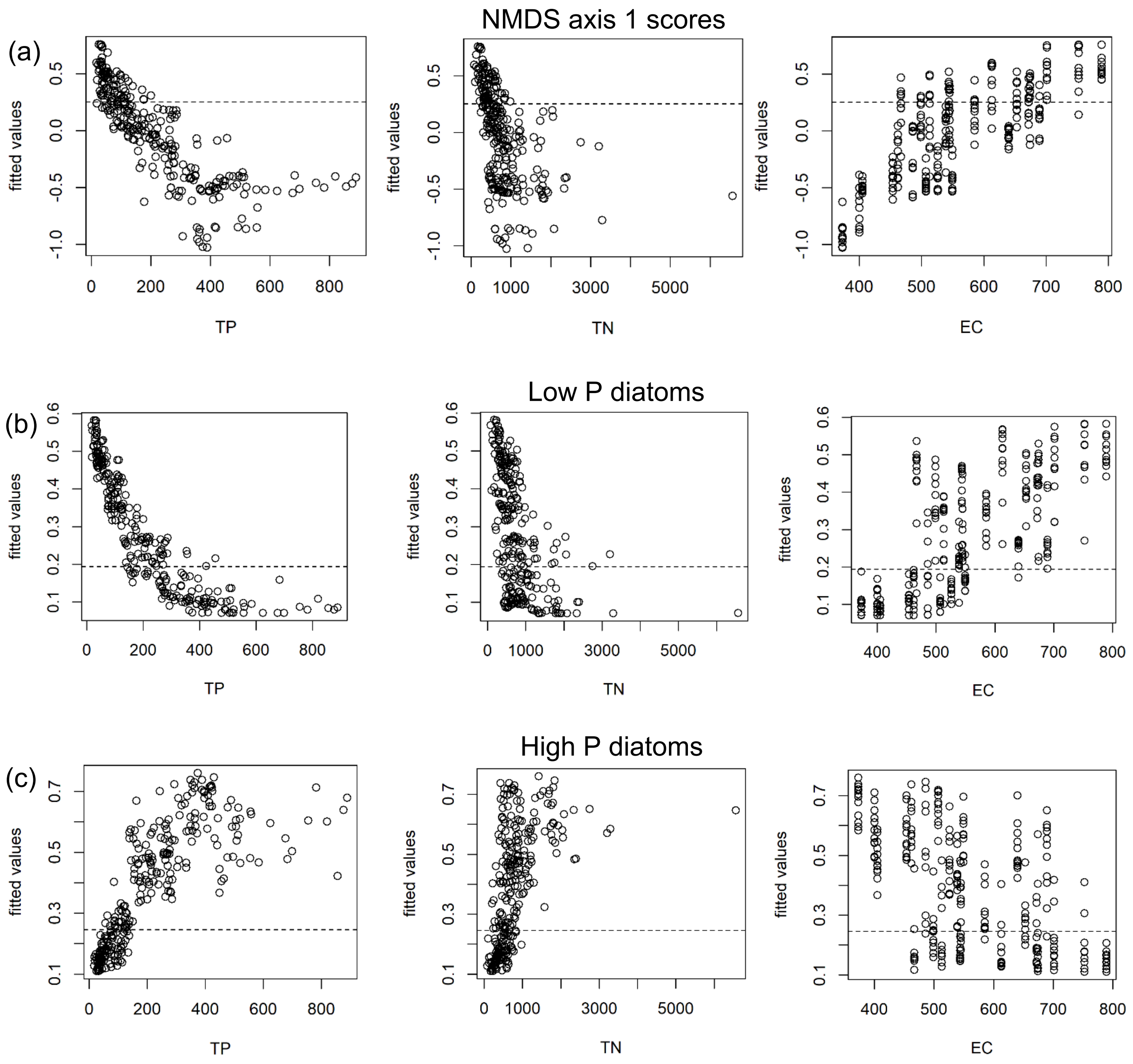
NMDS axis 1 scores
(a)
fitted values
TP
TN
EC
Low P diatoms
(b)
High P diatoms
(c)

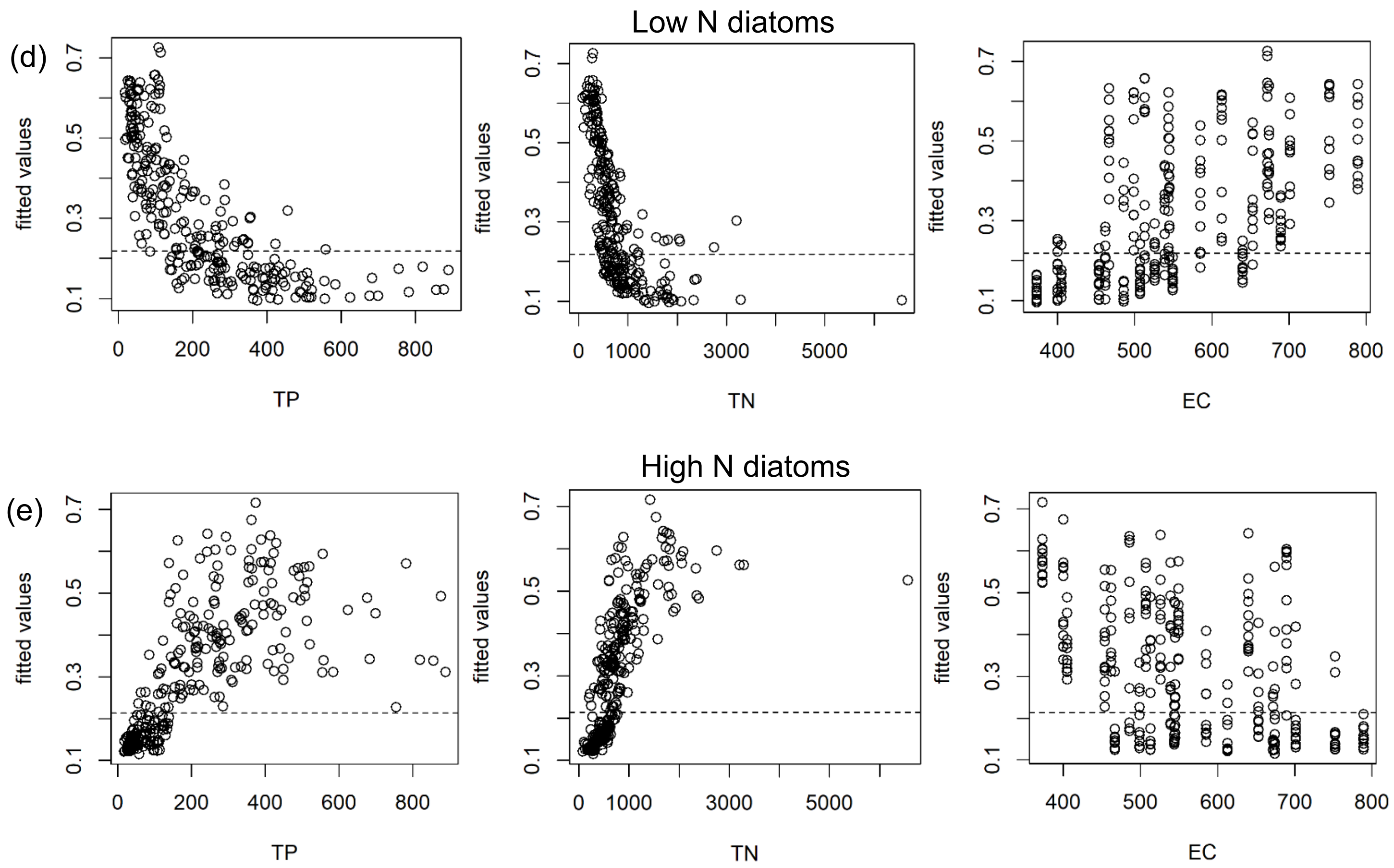


Figure S6. Plots of fitted values for nonmetric multidimensional (NMDS) axis 1 scores (a), and relative abundances of low phosphorus (b), high phosphorus (c), low nitrogen (d), and high nitrogen (e) diatom OTUs. EC = electrical conductivity.

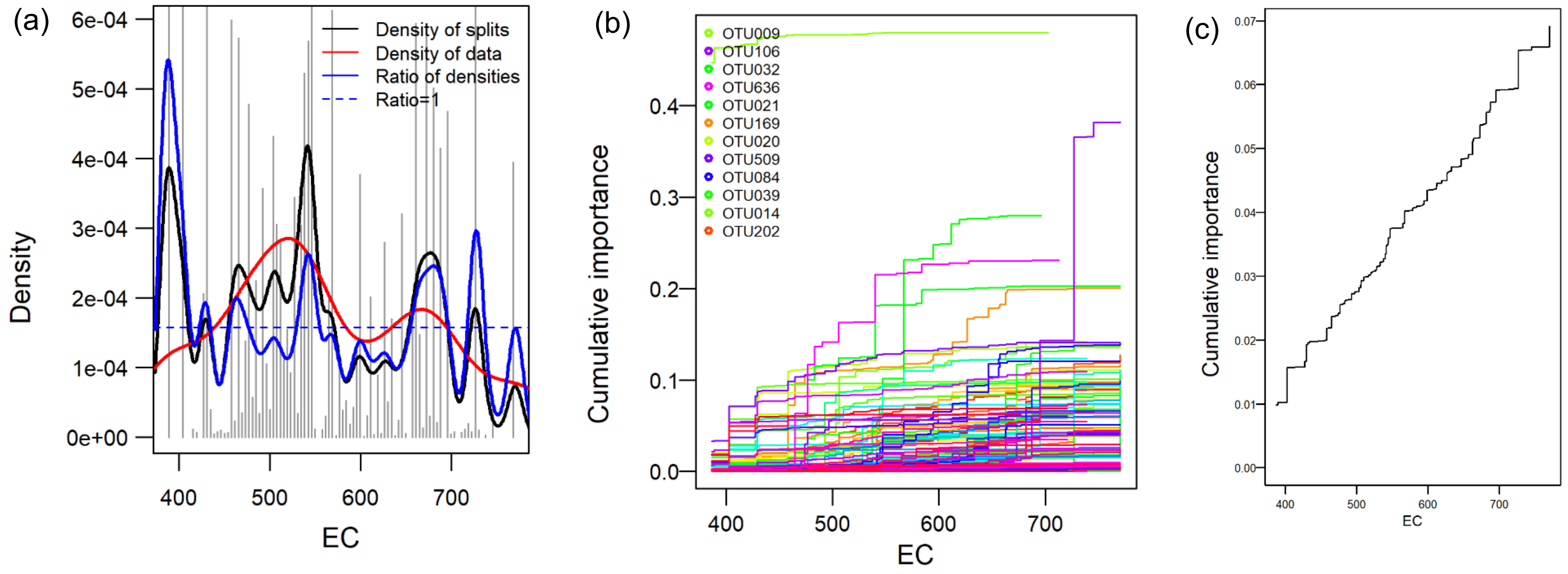


Figure S7. Conductivity results from gradient forest analysis showing splits density plots (a), cumulative importance plots for significant OTUs (b), and cumulative importance plots of assemblage change (c).

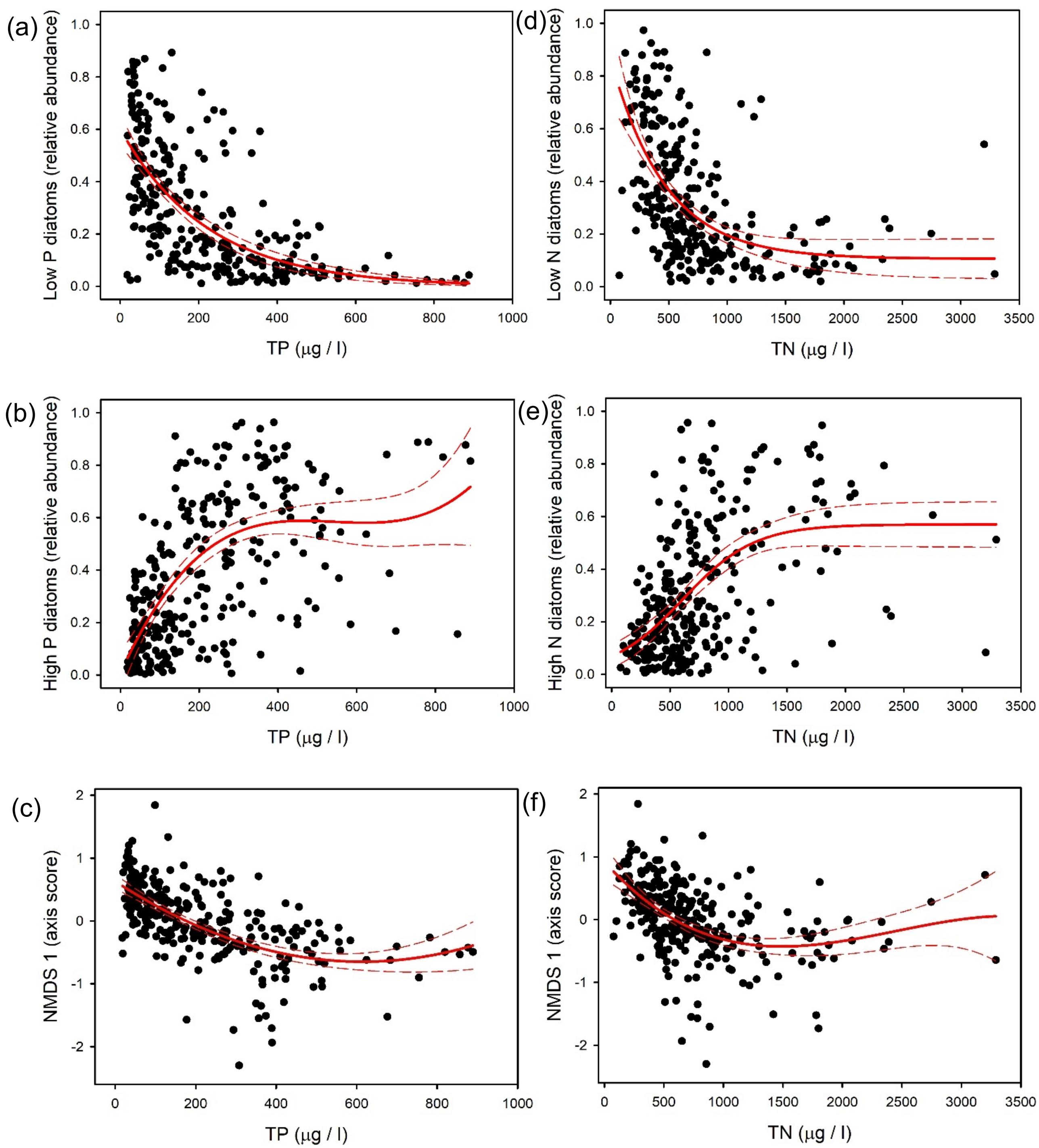


Figure S8. Scatter plots of raw data showing relationships of diatom metrics and nonmetric multidimensional scaling (NMDS) axis scores with TP (a–c) and TN (d–f) concentrations. Regression lines with 95% confidence intervals are simply intended to highlight relationships and were created using the regression wizard in SigmaPlot v. 14. Regressions: Low P–TP ($R^2$ =

0.44), High P–TP ($R^2 = 0.40$), NMDS 1–TP = ($R^2 = 0.41$), Low N–TN = ($R^2 = 0.35$), High N–TN = ($R^2 = 0.29$), NMDS 1–TN = ($R^2 = 0.24$).

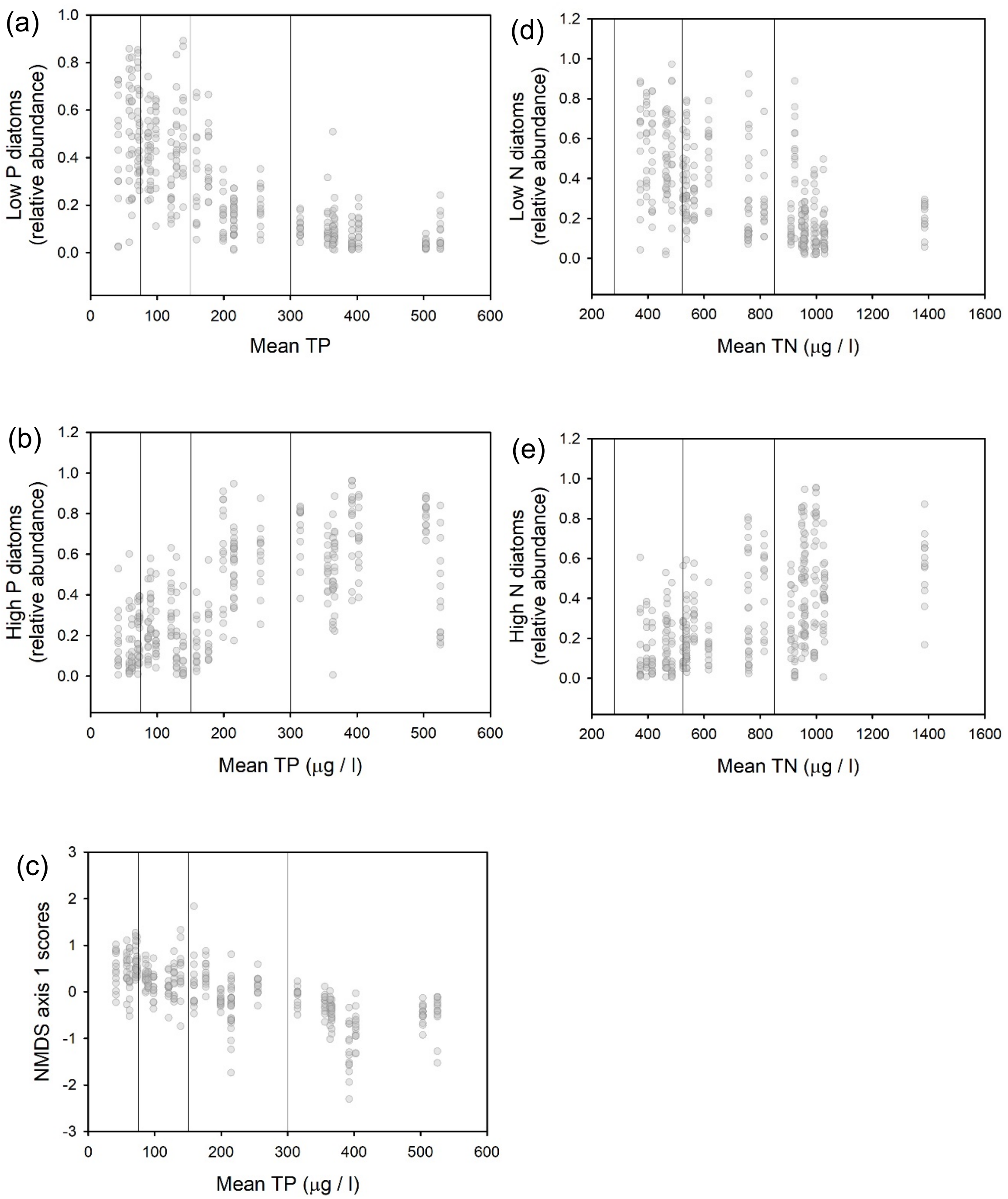


Figure S9. Within site variation of diatom metrics and nonmetric multidimensional scaling (NMDS) axis scores during the study as plotted by site means of TP (a–c) and TN (d–e) concentrations. Each vertical distribution of points is an individual site. When coupled with the variability in nutrient concentrations, these figures indicate the benefit of including all nutrient

and diatom observations to improve the robustness of data analyses. Vertical lines demarcate portions of TP and TN gradients within which numerous and large changes in diatom assemblages occurred as reported in the manuscript and as summarized in figure 6

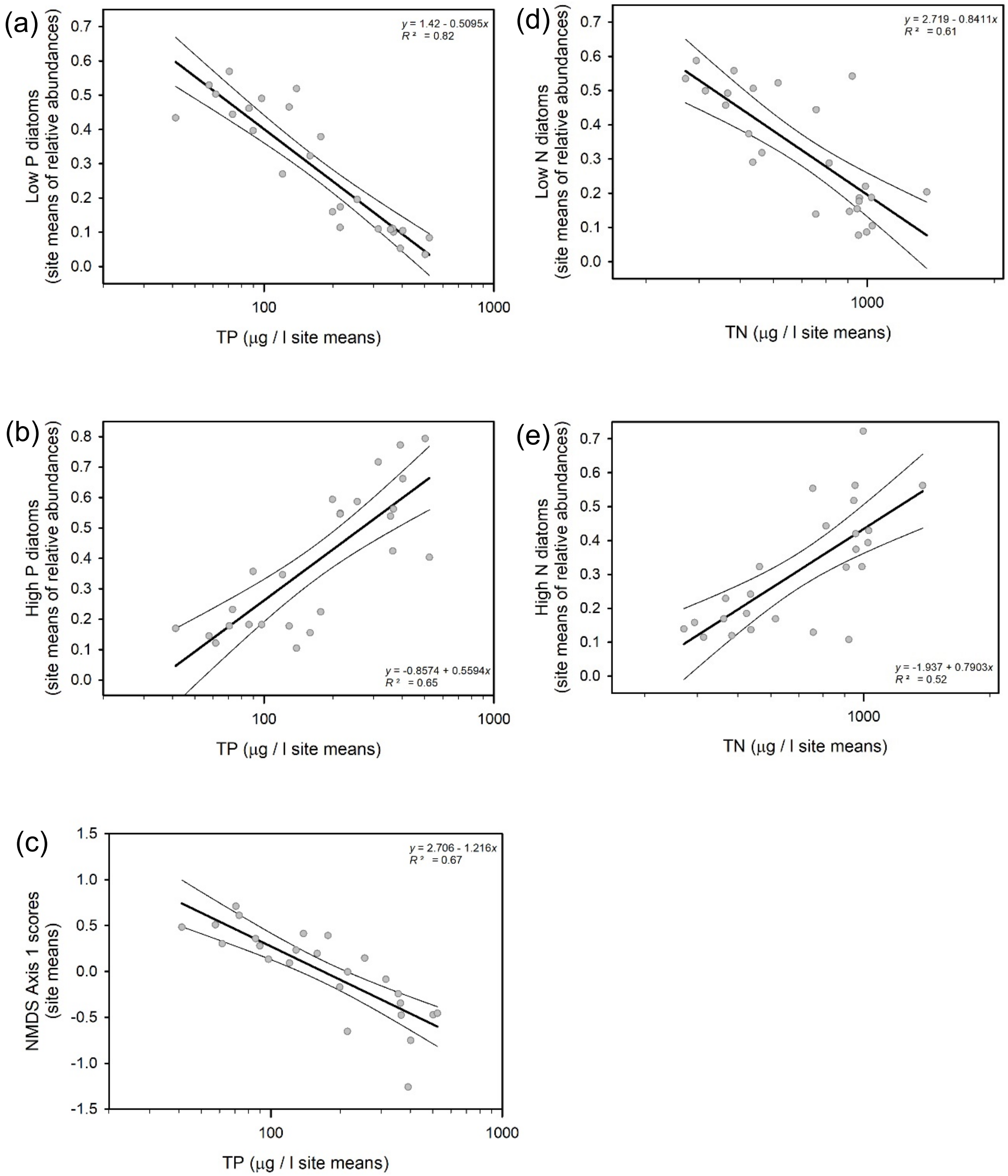


Figure S10. Linear regressions showing relationships between means of diatom metrics or nonmetric multidimensional (NMDS) axis 1 scores and means of TP (a–c) or TN (d–e) concentrations ($n$ = 25). These results provide further support for the robustness of diatom relationships with TP and TN.
