# Supplementary material for: DNA metabarcoding effectively quantifies diatom responses to nutrients in streams
Source: Ecol Appl. 2020 Aug 18;30(8):e02205. doi: 10.1002/eap.2205 (PMC7731896; doi:10.1002/eap.2205)
Supplement: Supplementary file 2 — Appendix S2 [file EAP-30-e02205-s002.pdf]

Supporting Information. Smucker, N.J., E.M. Pilgrim, C.T. Nietch, J.A. Darling, and B.R. Johnson. 2020. DNA metabarcoding effectively quantifies diatom responses to nutrients in streams. Ecological Applications.

Appendix S2

```
#This analysis uses Threshold Indicator Taxa Analysis v.2.1 (TITAN2) to examine if there are community changepoints in diatom species (OTUs based on rbcl sequencing) responses to total phosphorus and total nitrogen concentrations in the Little Miami River watershed.

#Install packages and load data
install.packages("TITAN2")
library(TITAN2)
install.packages("snow")

diatomsTP <- read.csv("diatomsTP.csv", header = TRUE, row.names=1)
diatomsTN <- read.csv("diatomsTN.csv", header = TRUE, row.names=1)
TP <- read.csv("TP.csv", header = TRUE, row.names=1)
TN <- read.csv("TN.csv", header = TRUE, row.names=1)

#Run TITAN2 for TP
LMR.titan <- titan(TP, diatomsTP, minSplt = 5, numPerm = 1000, boot = TRUE, nBoot = 1000,
imax = FALSE, ivTot = FALSE, pur.cut = 0.95, rel.cut = 0.95, ncpus = 68, memory = FALSE)

#For TITAN2 for TN

LMRTN.titan <- titan(TN, diatomsTN, minSplt = 5, numPerm = 1000, boot = TRUE, nBoot =
1000, imax = FALSE, ivTot = FALSE, pur.cut = 0.95, rel.cut = 0.95, ncpus = 68, memory =
FALSE)

#figures (just examples – aesthetics differ from final versions but data and results are the same)
tiff(file = "sumzTPfiltered.tiff", width = 7, height = 8, units = "in", res = 300)
plotSumz(LMR.titan, filter=TRUE, leg.x=1.1, leg.y=.99, bty="o", cex=1.2, cex.axis=1,
cex.leg=0.9, cex.lab=1.4, xlab=expression(paste("TP (", mu, "g/l)")), col1="steelblue2",
col2="red")
dev.off()

tiff(file = "TaxaTP.tiff", width = 7, height = 8, units = "in", res = 300)
plotTaxa(LMR.titan, filter=TRUE, leg.x=1.1, leg.y=5.5, bty="o", cex=.9, cex.axis=.9, cex.leg=1.2,
cex.lab=1.2, xlab=expression(paste("TP (", mu, "g/l)")), col1="steelblue2", fil1=NA, col2="red",
fil2=NA)
dev.off()

tiff(file = "sumzTNfiltered.tiff", width = 7, height = 8, units = "in", res = 300)
plotSumz(LMRTN.titan, filter=TRUE, leg.x=1.1, leg.y=.99, bty="o", cex=1.2, cex.axis=1,
cex.leg=0.9, cex.lab=1.4, xlab=expression(paste("TN (", mu, "g/l)")), col1="steelblue2",
col2="red")
dev.off()

tiff(file = "TaxaTN.tiff", width = 7, height = 8, units = "in", res = 300)
plotTaxa(LMRTN.titan, filter=TRUE, leg.x=1.1, leg.y=5.5, bty="o", cex=1, cex.axis=1,
cex.leg=1.2, cex.lab=1.2, xlab=expression(paste("TN (", mu, "g/l)")), col1="steelblue2", fil1=NA,
col2="red", fil2=NA)
dev.off()
```

```
#Code used for boosted regression trees

#Install packages and load data
install.packages("dismo")
install.packages("gbm")
library(dismo)
library(gbm)

diatoms <- read.csv("diatoms2.csv", header = TRUE, row.names=1)

set.seed(853) #random number generator used to set seed (0-1000)

#NMDS axis 1 BRT code
NMDS1.tc2.lr001 <- gbm.step(data=diatoms, gbm.x = 2:4, gbm.y = 6, family = "gaussian",
tree.complexity = 2, learning.rate = 0.001, bag.fraction = 0.5)

summary(NMDS1.tc2.lr001)

gbm.plot(NMDS1.tc2.lr001)
gbm.plot.fits(NMDS1.tc2.lr001)

#Low phosphorus diatom relative abundance BRT code
LP.tc2.lr001 <- gbm.step(data=diatoms, gbm.x = 2:4, gbm.y = 8, family = "gaussian",
tree.complexity = 2, learning.rate = 0.001, bag.fraction = 0.5)

summary(LP.tc2.lr001)

gbm.plot(LP.tc2.lr001)
gbm.plot.fits(LP.tc2.lr001)

#High phosphorus diatom relative abundance BRT code
HP.tc2.lr001 <- gbm.step(data=diatoms, gbm.x = 2:4, gbm.y = 7, family = "gaussian",
tree.complexity = 2, learning.rate = 0.001, bag.fraction = 0.5)

summary(HP.tc2.lr001)

gbm.plot(HP.tc2.lr001)
gbm.plot.fits(HP.tc2.lr001)

#Low nitrogen diatom relative abundance BRT code
LN.tc2.lr001 <- gbm.step(data=diatoms, gbm.x = 2:4, gbm.y = 10, family = "gaussian",
tree.complexity = 2, learning.rate = 0.001, bag.fraction = 0.5)

summary(LN.tc2.lr001)

gbm.plot(LN.tc2.lr001)
gbm.plot.fits(LN.tc2.lr001)

#High nitrogen diatom relative abundance BRT code
HN.tc2.lr001 <- gbm.step(data=diatoms, gbm.x = 2:4, gbm.y = 9, family = "gaussian",
tree.complexity = 2, learning.rate = 0.001, bag.fraction = 0.5)
```

```
summary(HN.tc2.lr001)

gbm.plot(HN.tc2.lr001, variable.no=0)
gbm.plot.fits(HN.tc2.lr001)
```

```
#Code used for gradient forest analysis

#Install packages and load data
install.packages("gradientForest", repos="http://r-forge.r-project.org")
library(gradientForest)

#Read in data
diatoms <- read.csv("diatoms2.csv", header = TRUE, row.names=1)
nutrientsEC <- read.csv("nutrientsEC.csv", header = TRUE, row.names=1)

#Run gradient forest analysis - "gfNObin" is final model - "gfNObin" includes conductivity
gfNObin <- gradientForest(cbind(nutrientsEC, diatoms),
      predictor.vars = colnames(nutrientsEC), response.vars = colnames(diatoms),
      ntree = 500, transform = NULL, compact = F, corr.threshold = 0.5)

#Plot variable importance
plot(gfNObin, plot.type = "O")

#Plot the R2 measure of the fit of random forest models for each species
plot(gfNObin, plot.type = "P", show.names = F, horizontal = F,
     cex.axis = 1, cex.labels = 0.7, line = 2.5)

#Returns R2 for all OTUs with R2>0
importance(gfNObin, type = c("Species"), sort = TRUE)

#For making figures
most_important <-names(importance(gfNObin))[1:3]
EC <-names(importance(gfNObin))[1]
TP <-names(importance(gfNObin))[2]
TN <-names(importance(gfNObin))[3]

#The following will plot split densities for all three variables at once
plot(gfNObin, plot.type = "S", imp.vars = most_important, mfrow = c(1,3), leg.posn = "topright",
      leg.panel = 2,  cex.legend = 0.9, cex=.5, cex.axis = 0.9, cex.lab = 1, line.ylab = 0,  las=1,
      par.args = list(mgp = c(2, 0.75, 0), mar = c(3, 3.5, 0.1, 1)))

#The following is to produce split densities plots for individual predictors
plot(gfNObin, plot.type = "S", imp.vars = TP, leg.posn = "topright",
     cex.legend = 0.9, cex=.5, cex.axis = 0.9, cex.lab = 1, line.ylab = 0,
     las=1, par.args = list(mgp = c(2, 0.75, 0), mar = c(3, 3.5, 0.1, 1)))

plot(gfNObin, plot.type = "S", imp.vars = TN, leg.posn = "topright",
     cex.legend = 0.9, cex=.5, cex.axis = 0.9, cex.lab = 1, line.ylab = 0,
     las=1, par.args = list(mgp = c(2, 0.75, 0), mar = c(3, 3.5, 0.1, 1)))

plot(gfNObin, plot.type = "S", imp.vars = EC, leg.posn = "topright",
     cex.legend = 0.9, cex=.5, cex.axis = 0.9, cex.lab = 1, line.ylab = 0,
     las=1, par.args = list(mgp = c(2, 0.75, 0), mar = c(3, 3.5, 0.1, 1)))

#Plot cumulative importance plots of significant OTUs for all three predictors at once
plot(gfNObin, plot.type = "C", imp.vars = most_important, mfrow = c(1,3), las = 1,
```

```
    show.overall = F, common.scale = TRUE, legend = T, leg.posn = "topleft",
    leg.nspecies = 12, cex.lab = 1, cex.legend = 0.7, cex.axis = 0.8,
    line.ylab = 0.5, par.args = list(mgp = c(1.5,0.5, 0),
    mar = c(2.5, 2, 0.5, 0.5), omi = c(0, 0.3, 0, 0)))

#Plot cumulative importance plots of significant OTUs for individual predictors
plot(gfNObin, plot.type = "C", imp.vars = TP, mfrow = c(1,3), las = 1,
    show.overall = F, common.scale = TRUE, legend = T, leg.posn = "topleft",
    leg.nspecies = 12, cex.lab = 1, cex.legend = 0.7, cex.axis = 0.8,
    line.ylab = 0.5, par.args = list(mgp = c(1.5,0.5, 0),
    mar = c(2.5, 2, 0.5, 0.5), omi = c(0, 0.3, 0, 0)))

plot(gfNObin, plot.type = "C", imp.vars = TN, mfrow = c(1,3), las = 1,
    show.overall = F, common.scale = TRUE, legend = T, leg.posn = "topleft",
    leg.nspecies = 12, cex.lab = 1, cex.legend = 0.7, cex.axis = 0.8,
    line.ylab = 0.5, par.args = list(mgp = c(1.5,0.5, 0),
    mar = c(2.5, 2, 0.5, 0.5), omi = c(0, 0.3, 0, 0)))

plot(gfNObin, plot.type = "C", imp.vars = EC, mfrow = c(1,3), las = 1,
    show.overall = F, common.scale = TRUE, legend = T, leg.posn = "topleft",
    leg.nspecies = 12, cex.lab = 1, cex.legend = 0.7, cex.axis = 0.8,
    line.ylab = 0.5, par.args = list(mgp = c(1.5,0.5, 0),
    mar = c(2.5, 2, 0.5, 0.5), omi = c(0, 0.3, 0, 0)))

#Plot cumulative importance plots of assemblage change for all three predictors at once
plot(gfNObin, plot.type = "C", imp.vars = most_important, mfrow = c(1,3), las = 1, show.species
     = F, common.scale = T, cex.axis = 0.8,
    cex.lab = 1, line.ylab = 0.5, par.args = list(mgp = c(1.5, .5, 0),
    mar = c(2.5, 2, 0.5, 0.5), omi = c(0, 0.3, 0, 0)))

#Plot cumulative importance plots of assemblage change for individual predictors

plot(gfNObin, plot.type = "C", imp.vars = TP, las = 1, show.species = F, common.scale = T,
     cex.axis = 0.8,  cex.lab = 1, line.ylab = 0.5, par.args = list(mgp = c(1.5, .5, 0),  mar = c(2.5,
     2, 0.5, 0.5), omi = c(0, 0.3, 0, 0)))

plot(gfNObin, plot.type = "C", imp.vars = TN, las = 1, show.species = F, common.scale = T,
     cex.axis = 0.8,  cex.lab = 1, line.ylab = 0.5, par.args = list(mgp = c(1.5, .5, 0),  mar = c(2.5,
     2, 0.5, 0.5), omi = c(0, 0.3, 0, 0)))

plot(gfNObin, plot.type = "C", imp.vars = EC, las = 1, show.species = F, common.scale = T,
     cex.axis = 0.8,  cex.lab = 1, line.ylab = 0.5, par.args = list(mgp = c(1.5, .5, 0),  mar = c(2.5,
     2, 0.5, 0.5), omi = c(0, 0.3, 0, 0)))
```
